# SOX2 commands LIM homeobox transcription factors in choroid plexus development and tumorigenesis

**DOI:** 10.1093/neuonc/noaf085

**Published:** 2025-03-25

**Authors:** Lukas J Faltings, Fengjuan Lin, Mariam Zahran, Heena Jalili, Anjali Siluveru, Mahek Chaudry, Leah M Wachsmuth, Navjot Guru, Melanie Schoof, Ping Cao, Yuan Huang, Noreen Mian, Sohyun Moon, Asmaa Zahran, Kristen Green, Siddhi Modi, Maheen Umer, James Q Virga, Ying-Tao Zhao, Ulrich Schüller, Qun Li, Haotian Zhao

**Affiliations:** Department of Biomedical Sciences, New York Institute of Technology College of Osteopathic Medicine, Old Westbury, New York, USA; Department of Oncology, Shanghai East Hospital, Tongji University School of Medicine, Shanghai, People’s Republic of China; Department of Biomedical Sciences, New York Institute of Technology College of Osteopathic Medicine, Old Westbury, New York, USA; Department of Biomedical Sciences, New York Institute of Technology College of Osteopathic Medicine, Old Westbury, New York, USA; Department of Biomedical Sciences, New York Institute of Technology College of Osteopathic Medicine, Old Westbury, New York, USA; Department of Biomedical Sciences, New York Institute of Technology College of Osteopathic Medicine, Old Westbury, New York, USA; Department of Biomedical Sciences, New York Institute of Technology College of Osteopathic Medicine, Old Westbury, New York, USA; Department of Biomedical Sciences, New York Institute of Technology College of Osteopathic Medicine, Old Westbury, New York, USA; Institute for Neuropathology, University Medical Center Hamburg-Eppendorf, Hamburg, Germany; Research Institute Children’s Cancer Center, Hamburg, Germany; Department of Biomedical Sciences, New York Institute of Technology College of Osteopathic Medicine, Old Westbury, New York, USA; Department of Biomedical Sciences, New York Institute of Technology College of Osteopathic Medicine, Old Westbury, New York, USA; Department of Biomedical Sciences, New York Institute of Technology College of Osteopathic Medicine, Old Westbury, New York, USA; Department of Biomedical Sciences, New York Institute of Technology College of Osteopathic Medicine, Old Westbury, New York, USA; Department of Biology, College of Arts and Science, Adelphi University, Garden City, New York, USA; Department of Biomedical Sciences, New York Institute of Technology College of Osteopathic Medicine, Old Westbury, New York, USA; Department of Biomedical Sciences, New York Institute of Technology College of Osteopathic Medicine, Old Westbury, New York, USA; Department of Biomedical Sciences, New York Institute of Technology College of Osteopathic Medicine, Old Westbury, New York, USA; Department of Biomedical Sciences, New York Institute of Technology College of Osteopathic Medicine, Old Westbury, New York, USA; Department of Biomedical Sciences, New York Institute of Technology College of Osteopathic Medicine, Old Westbury, New York, USA; Institute for Neuropathology, University Medical Center Hamburg-Eppendorf, Hamburg, Germany; Research Institute Children’s Cancer Center, Hamburg, Germany; Department of Oncology, Shanghai East Hospital, Tongji University School of Medicine, Shanghai, People’s Republic of China; Department of Biomedical Sciences, New York Institute of Technology College of Osteopathic Medicine, Old Westbury, New York, USA

**Keywords:** choroid plexus tumor, LMX1A, LMX1B, NOTCH, SOX2

## Abstract

**Background:**

Choroid plexus (CP) tumors are rare brain neoplasms that mainly affect the pediatric population. Unlike benign CP papilloma (CPP), CP carcinoma (CPC) is an aggressive cancer with a dismal survival rate. Despite chromosome-wide rearrangements, drivers of most CP tumors remain elusive except recurrent alterations in *TP53*. Studies of signaling dysregulation may bring biological understanding of these malignancies. Previous studies implicated NOTCH signaling in CP tumors; we developed mouse models of CP tumors driven by NOTCH activation and *Trp53* loss, respectively. This work examined the role of the transcription factor SOX2 in CP development and tumorigenesis.

**Methods:**

Multi-omics approaches were used to characterize cellular heterogeneity in NOTCH-driven CP tumors. SOX2 functions in the molecular signature of tumor cells were investigated.

**Results:**

Single-cell transcriptomics and epigenetics methods identified diverse cell populations in tumors that resemble normal CP, such as epithelial and glial groups. Pseudotime trajectory analysis indicated that NOTCH-driven CP tumor arises from bipotential glial progenitors and retains a progenitor-like signature characterized by an enhanced SOX2 profile. SOX2 inactivation attenuated progenitor-like features and blunted tumor growth. Integrative omics studies revealed SOX2 binding to genes expressed in progenitors in the rhombic lip, including LIM homeobox transcription factors LMX1A and LMX1B. Consistently, SOX2 maintains progenitor identity through regulating their expression in CP tumors and during development, whereas LMX1A and LMX1B support SOX2 functions in tumor cell proliferation. Furthermore, spatial transcriptomics revealed aberrant SOX2 and LMX1A expression in human CP tumors.

**Conclusions:**

SOX2-LMX1 signaling maintains progenitor identity in CP development and tumor formation.

## Graphical Abstract

**Graphical Abstract:**
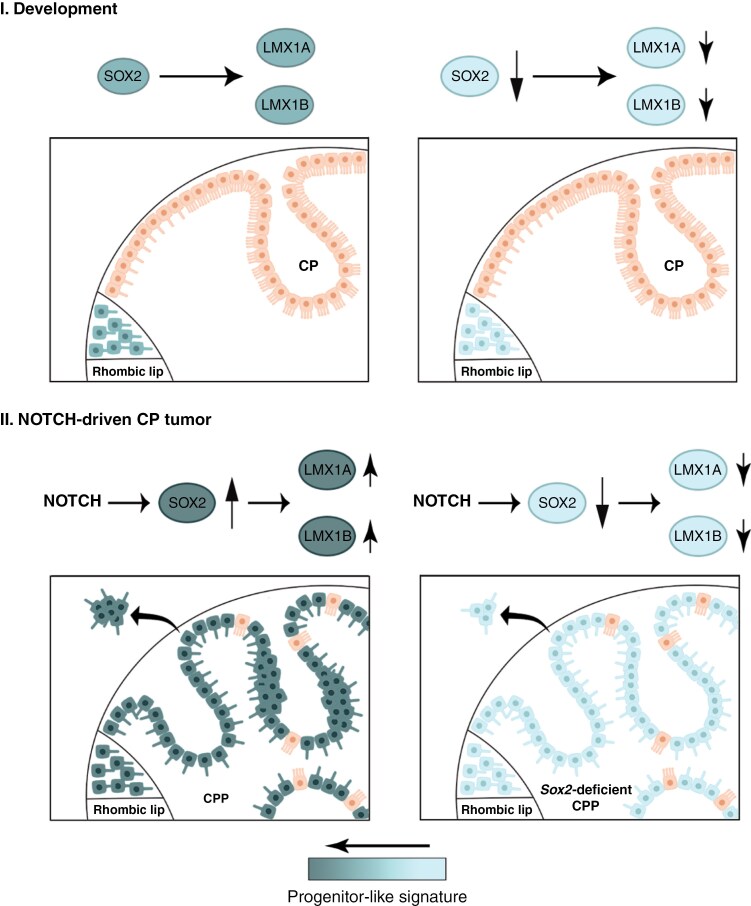

**Key Points**

1. SnRNA-seq and scATAC-seq data revealed diverse cell populations in NOTCH-driven CP tumors characterized by a progenitor signature.
2. SOX2-LMX1 signaling is required for the progenitor signature in NOTCH-driven CP tumor development.

**Importance of the Study**

Choroid plexus (CP) tumors are rare brain neoplasms that mostly occur in childhood. Despite frequent chromosomal alterations and recurrent mutations in *TP53*, the bland mutational profile indicates a lack of driver alterations. We hypothesize that CP tumors are driven by transcriptional and epigenetic changes associated with chromosome-wide rearrangements. Consistent with abnormal NOTCH signaling in a subset of CP tumors, snRNA-seq and scATAC-seq data unraveled cellular diversity in NOTCH-driven CP tumor in mice. Cell trajectory analysis showed that tumor arises from bipotential progenitors in the rhombic lip, while displaying a progenitor-like signature characterized by aberrant expression of SOX2. Multi-omics studies demonstrated that SOX2 acts downstream of NOTCH, and recruits transcription factors LMX1A/B to maintain the molecular and epigenetic landscape of progenitors during CP development and tumorigenesis. Accordingly, spatial transcriptomics detected abnormal SOX2 and LMX1A expression in CP tumors in humans. Targeting SOX2-LMX1 signaling may provide a therapeutic venue for CP tumors.

The choroid plexus (CP) is an epithelial tissue lining brain ventricles that produces the majority of the cerebrospinal fluid (CSF).^1^ The multifaceted secretory tissue is composed of an outer layer of epithelial cells that encase a vascular stroma to form the blood-CSF barrier.^2^ The CP consists of multiple cell types to carry out its functions, including endothelial cells, fibroblasts, neurons, and macrophages that display distinct transcriptional programs across ages and ventricles in mouse brain.^3^

The CP arises from the germinal centers in the dorsal roof plate, telencephalic cortical hem, and the rhombic lip in the hindbrain during development.^4,5^ These progenitors are located along the base of the CP proximal to the brain and express markers such as R-spondin (*Rspo*) and *Msx* genes.^3,6^ Cell fate tracing experiments have demonstrated that the hindbrain CP is derived from progenitor cells in the rhombic lip that express growth differentiation factor 7 (GDF7) and LIM homeobox transcription factor 1 alpha (LMX1A).^7–9^ The differentiation of CP epithelium is coordinated by a group of transcription regulators including zinc finger protein 423 (ZFP423), LMX1A, and orthodenticle homeobox 2 (OTX2).^9–12^ Previous studies demonstrated important roles of WNT, NOTCH, bone morphogenetic proteins (BMPs), and Sonic Hedgehog (SHH) signaling in CP morphogenesis.^8,13–16^ Recent single-cell and lineage studies revealed that a glial progenitor pool gives rise to epithelial and neuronal cells in the CP.^3^ Differentiating CP epithelial cells next to progenitor domains undergo an intermediate stage of multiciliation, a process directed by Geminin Coiled-Coil Domain Containing (GMNC), TAp73, a *Trp53* homolog, and forkhead box J1 (FOXJ1).^3,17,18^ Mature epithelial cells turn on the expression of genes involved in CSF production and barrier functions, such as water transporter aquaporin-1 (AQP1), and transthyretin (TTR).^12,19^

Tumors of the CP are a rare and heterogeneous group of neoplasms that predominantly occur in pediatric patients.^20^ Histologically, CP tumors consist of CP papilloma (CPP, WHO grade 1), atypical CP papilloma (aCPP, WHO grade 2), and CP carcinoma (CPC, WHO grade 3). CPPs are mostly treated by surgery alone with excellent prognosis; however, CPC is an aggressive brain cancer frequently associated with poor outcome and risk for recurrence and metastasis.^21,22^ Although chemotherapy is typically used in the treatment of CPC, older patients may be recommended to receive irradiation. Survivors often suffer from debilitating long-term treatment effects, whereas optimal treatment for recurrent CPC is not established.^22,23^

Though chromosome rearrangements are frequently observed in CP tumors, no consistent drivers have been identified except recurrent mutations in *TP53* in CPCs.^24–26^ DNA methylation profiling categorizes CP tumors into 3 subgroups that may provide prognostic information, whereas CPP and aCPP exhibit no significant differences in gene expression, methylation, or chromosome-wide alterations.^27^ Previous studies showed aberrant signaling activities in CP tumors.^26,28,29^ Animal models were developed to evaluate the contribution of defective *Trp53*, NOTCH and SHH signaling, *Myc* oncogene, Wnt signaling, and defective multiciliation in CP tumors.^17,30–34^ SRY-Box Transcription Factor 2 (SOX2) is a master regulator in ontogenesis, stem cell reprogramming, and cancer.^35^ Accumulating evidence suggests that SOX2 promotes cancer stemness, drives tumor initiation, and contributes to drug resistance and poor survival of cancer patients. A recent report showed that increased SOX2 expression is associated with high-grade CP tumors.^36^

In this study, single-nucleus RNA sequencing (snRNA-seq) and single-cell Assay for Transposase-Accessible Chromatin using sequencing (scATAC-seq) showed that NOTCH-driven CP tumors partitioned into clusters identified as epithelial-like, mesenchymal, endothelial, immune, neuronal, and glia-like cells, similar to populations in normal CP in mice.^3^ Though tumor cells reside in an epithelial-like compartment, they arise from binary glial progenitors and exhibit an enrichment of genes associated with glial progenitors in the rhombic lip, including *Sox2*, *Zfp423*, and *Rspo1*.^3,10^*Sox2* deletion reduced progenitor-like profiles, decreased tumor cell proliferation, and blocked tumor development. Chromatin immunoprecipitation (ChIP) and scATAC-seq studies revealed collaborative SOX2 occupancy of promoters of genes expressed in the progenitors in the rhombic lip and the hindbrain. In agreement, SOX2 is critical in regulating the expression of LMX1A and LMX1B in tumor cells as well as progenitors in the rhombic lip, whereas LMX1A and LMX1B mediate SOX2 functions in tumor cells. Spatial transcriptomics studies showed that a subset of human CP tumors exhibits abnormal SOX2 and LMX1A expression. Therefore, targeting aberrant SOX2-LMX1 signaling may provide therapeutic strategies in CP tumors.

## Materials and Methods

### Animals

*Gt(ROSA)26Sor* ^*tm*^1^*.Notch1Dam*^*/J* (*Rosa26-NICD1*) mice, B6N.129-*Ptch1*^*tm1Hahn*^/J (*Ptch*^*flox/flox*^) mice, *Sox2*^*tm*^1^*.1Lan*^/J (*Sox2*^*flox/flox*^) mice, B6.129P2-*Trp53*^*tm1Brn*^/J (*Trp53*^*flox/flox*^) mice, *Rb1*^*tm2Brn*^/J (*Rb*^*flox/flox*^) mice, and *C57BL/6* mice (all from Jackson Laboratory), and *Tg(Lmx1a-cre)1Kjmi* (*Lmx1a-Cre*) mice were maintained by breeding with C57BL/6 mice. All animal experimental procedures were approved by Institutional Animal Care and Use Committee and performed in compliance with national regulatory standards.

### Human Samples

CP specimens were procured with informed consent from patients following the requirements by institutional review boards. Diagnoses of human CP specimens were reviewed using standard WHO criteria.^20^

### Cell Culture and Viruses

NOTCH-driven CP tumors, *Rb1/Trp53*-deficient CPC cells, and CCHE human CPC cells were cultured as described previously.^17,29,33^ Human CP organoid was differentiated from the IMR-90 stem cell line (WiCell Research Institute, Inc.) as described previously.^37^ RBPJ Inhibitor-1 (RIN-1) (MedChemExpress) was used to treat tumor cells. Viruses expressing HA-tagged LMX1A or LMX1B (269200540200, 269210540200, Applied Biological Materials Inc.), and viruses expressing SOX2, shRNAs against *Sox2* (shADV-272885) or *Lmx1a* (shADV-263539, all from Vector Biolabs) were amplified and purified from AD-293 cells (Agilent Technologies).

### Immunohistochemistry, Immunofluorescence, Co-immunoprecipitation, and Immunoblotting

Immunostaining and co-immunoprecipitation were carried out as previously described.^33^ Primary antibodies used: anti-SOX2 (ab97959, Abcam; 66411-1-Ig, Proteintech), anti-LMX1A (ZRB1373, Millipore Sigma), anti-LMX1A (sc-134990, Santa Cruz Biotechnology, Inc.), anti-ARL13B (clone N295B/66, NeuroMab), anti-GFP (GFP-1010, Aves Lab), anti-Ki-67 (ab16667, Abcam), anti-HA (66006-1-Ig, Proteintech), anti-Aquaporin-1 (ab9566, Abcam; AB2219, Millipore Sigma), anti-OTX2 (AF1979, R&D Systems, Inc.), anti-β-Actin (A5441, Millipore Sigma), anti-MSX1 (4G1-c, Developmental Studies Hybridoma Bank), and anti-LMX1B (ab259926, Abcam).

### Microscopy and Image Analysis

Whole-mount bright field was obtained using a Nikon SMZ1000 Stereomicroscope (Nikon Instruments).^33^ Light and fluorescent microscopic images were obtained using a Zeiss AXIO Observer 7 Inverted Fluorescence Motorized XYZ Definite Focus Microscope, a ZEISS LSM 980 with Airyscan 2 confocal microscope, and a ZEISS Axioscan 7 as a slide scanner (Carl Zeiss Microscopy, LLC). Signal intensity data were quantified from fluorescence images using ImageJ software including the plug-in EzColocalization for single-cell fluorescence intensity measurement.^38,39^

### RT-qPCR, RNAscope, and CosMx Studies

All reactions were run on a QuantStudio 3 Real-Time PCR System (Thermo Fisher Scientific). Gene-specific primers and probes were used (Integrated DNA Technologies, Inc.).^33^ For RNAscope, RNAscope 2.5 HD Duplex Reagent was used according to the manufacturer’s instructions (Advanced Cell Diagnostics).^17^ High-plex in situ analysis of human sample was performed using CosMx Spatial Molecular Imager (NanoString).

### ChIP and Sequencing

ChIP was performed as previously described with anti-SOX2 (39843, Active Motif), anti-OTX2 (13497-1-AP, Proteintech), and anti-histone H3k27Ac antibodies (39133, Active Motif).^40^ SnRNA-seq and scATAC-seq were performed using the 10x Genomics Chromium single-cell gene expression profiling platform (10x Genomics). RNA-seq experiments and data analysis were performed as described previously.^33,41,42^

### Statistical Analysis

Information on experiment replication is provided in the legends. Statistical analyses were performed with GraphPad Prism 9.0 (GraphPad Software Inc.). All pooled data were expressed as the mean ± standard error of the mean (SEM). Variation within each group of data was examined based on the differences between each data point and the mean of the group. The Kolmogorov–Smirnov test was used to test the normal distribution of the data. Differences between 2 groups were compared using the paired *t* test or the unpaired 2-tailed *t* test. Differences between multiple groups were analyzed with ANOVA followed by Tukey’s multiple comparisons test. Results were considered significant at *P < 0.05; **P < 0.01; ***P < 0.001; ****P < 0.0001.

### Accession Numbers

Sequencing data are available from NCBI. Published single-cell sequencing data of the CP was analyzed (Gene Expression Omnibus [GEO] database GSE168704).^3^ RNA-seq data (BioProject ID, PRJNA282889) was analyzed.

## Results

### snRNA-Seq Data Reveals Dynamic Differentiation in NOTCH-Driven CP Tumors Characterized by a Glial Progenitor-Like Signature

**Figure 1.**
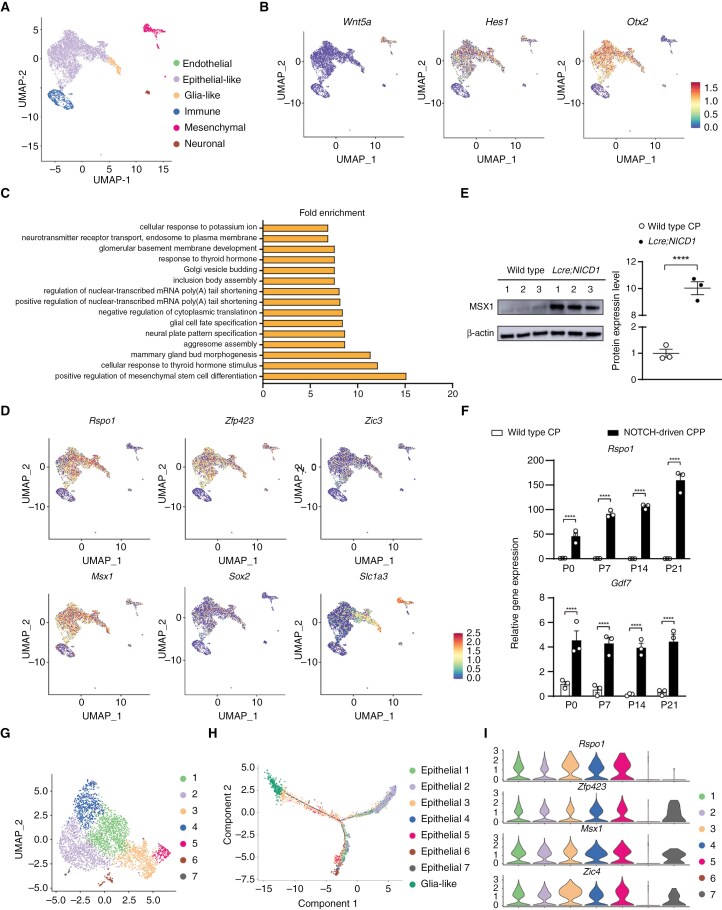
**SnRNA-seq reveals cellular diversity and dynamic differentiation in NOTCH-driven CP tumors characterized by a glial progenitor-like signature.** (A) Major cell types of a NOTCH-driven CPP from an adult *Lcre;NICD1* mouse. Uniform Manifold Approximation and Projection (UMAP) of 6428 single-nucleus profiles, colored by post hoc annotated cell type; also see Figure S1. (B) UMAP showing mesenchymal, epithelial, and NOTCH-activated profiles from snRNA-seq in NOTCH-driven CPP, colored by expression of Wnt5a (mesenchymal marker, *left*), *Hes1* (NOTCH pathway target, *middle*), and *Otx2* (CP epithelial marker, *right*); also see Figure S1. (C) GO analysis of differentially expressed genes in the epithelial-like tumor cell compartment in NOTCH-driven CPP. (D) The expression of markers for glial progenitors in the rhombic lip in NOTCH-driven CPP. UMAP shows 6428 single-nucleus profiles from snRNA-seq in NOTCH-driven CPP, colored by expression of genes associated with glial progenitors in the rhombic lip in the hindbrain (*Rspo1*, *Zfp423*, *Zic3*, *Msx1*, *Sox2*, and *Slc1a3*). (E) Western blot analysis of MSX1 expression in the CP of wild-type mice, and NOTCH-driven CPP in *Lcre;NICD1* animals (*n* = 3 per genotype, mean ± SEM, 2-tailed unpaired *t* test, ****P < 0.0001). Data were generated from 2 independent experiments. (F) RT-qPCR analysis of *Rspo1* and *Gdf7* mRNA levels in NOTCH-driven CPP and wild-type CP (*n* = 3 per time point per genotype, mean ± SEM, 2-tailed unpaired *t* test, ****P < 0.0001). Three independent experiments were conducted. (G) UMAP showing single-nucleus profiles in the epithelial-like compartment in (A) colored by subgroups; also see Figure S1. (H) Cell trajectory analysis of single-nucleus profiles of subgroups of the epithelial-like compartment, and the glia-like compartment in NOTCH-driven CPP. (I) Violin plots for the expression of *Rspo1*, *Zfp423*, *Msx1*, and *Zic4* in subgroups of the epithelial-like compartment in NOTCH-driven CPP.

To characterize the cellular architecture of CP tumors, snRNA-seq was conducted in NOTCH-driven CP tumors using a mouse model. Here, the *Rosa26-NICD1* mouse strain was used that exhibits Cre-mediated expression of the intracellular domain of NOTCH1 (NICD1) and green fluorescent proteins (GFP).^17^ After crossing to *Lmx1a-Cre* mice that express *Cre* in the rhombic lip and the CP, *Lmx1a-Cre;Rosa26-NICD1* (*Lcre;NICD1*) mice developed CPP.^33^ Uniform manifold approximation and projection (UMAP) applied to 6428 single-nucleus profiles in a NOTCH-driven CPP revealed cell types similar to those in the CP, including endothelial cells, epithelial-like cells, glia-like cells, immune cells, mesenchymal cells, and neuronal cells (Figure 1A, Figure S1A, Table S1). A subset of cells expressed neuronal markers *Neurod1/2*, whereas *Wnt5a* expression was detected in the mesenchymal cells (Figure S1B, Figure 1B, Table S1).^3,13^ Consistent with NOTCH activation, the expression of NOTCH target *Hes1* was enriched in the *Otx2*^*+*^ epithelial-like compartment (Figure 1B, Table S1). Copy number variation (CNV) estimation was increased in this cluster (Figure S1C, S1D), indicating that tumor cells reside in the epithelial-like group. Among 86 genes expressed in CP epithelial cells identified previously, 29 were detected in the epithelial-like compartment (Table S1).^3^ Subsequently, analysis of previously published RNA sequencing (RNA-seq) data of NOTCH-driven CPP was conducted. These studies showed that the majority (54 out of 86) of CP epithelial markers were significantly reduced in tumor cells by 3 weeks after birth.^17,33^ Consistently, RT-qPCR data revealed decreased *Ttr* and *Otx2* mRNA levels in tumor cells (Figure S1E). Taken together, these results indicate defective epithelial maturation in NOTCH-driven CP tumors.

There were 24 genes identified by previous studies as associated with glial progenitors that were detected in the glia-like cell compartment (Table S1). Interestingly, gene ontology (GO) analysis of the epithelial-like compartment revealed enrichment of the program of glial cell fate specification and neural plate pattern specification (Figure 1C). Indeed, expression of markers of progenitor in the rhombic lip, such as *Rspo1*, *Zfp423*, *Gdf7*, *Msx1*, *Zic3*, *Slc1a3*, and *Sox2*, was not only present in the glia-like compartment, but also detected in the epithelial-like cell group (Figure 1D, Table S1). Consistently, results from western blot and RT-qPCR studies showed significantly increased expression of these glial progenitor markers in tumor cells (Figure 1E, F). Moreover, analysis of RNA-seq data from NOTCH-driven CP tumors revealed increased expression of over 40 genes that are enriched in the glial population of the CP, including *Rspo1*, *Rspo3*, *Gdf7*, *Zic1-4*, *Slc1a3*, and *Sox2* (Table S2).^3^

The ectopic glial progenitor-like features in the tumor cells of the epithelial-like compartment suggested a developmental origin and/or dynamic differentiation in NOTCH-driven CP tumors. Accordingly, subclustering identified 7 subgroups in the epithelial-like compartment (Figure 1G, Figure S1F). Cell trajectory analysis arranged the glia-like compartment at 1 end, and 2 subgroups (#2 and #6) segregated at the distal ends of 2 branches, with another subgroup (#3) extending from the glia-like group toward the converging point of the branches (Figure 1H). All subgroups displayed *Otx2* expression, whereas most subgroups maintained aberrant expression of the glial progenitor markers (Figure 1I). Together, single-nucleus transcriptomics and gene expression studies revealed dynamic differentiation patterns in NOTCH-driven CP tumors that adopt a glial progenitor-like signature.

### SOX2 Is Expressed in CP Tumor in Humans and Mice

**Figure 2.**
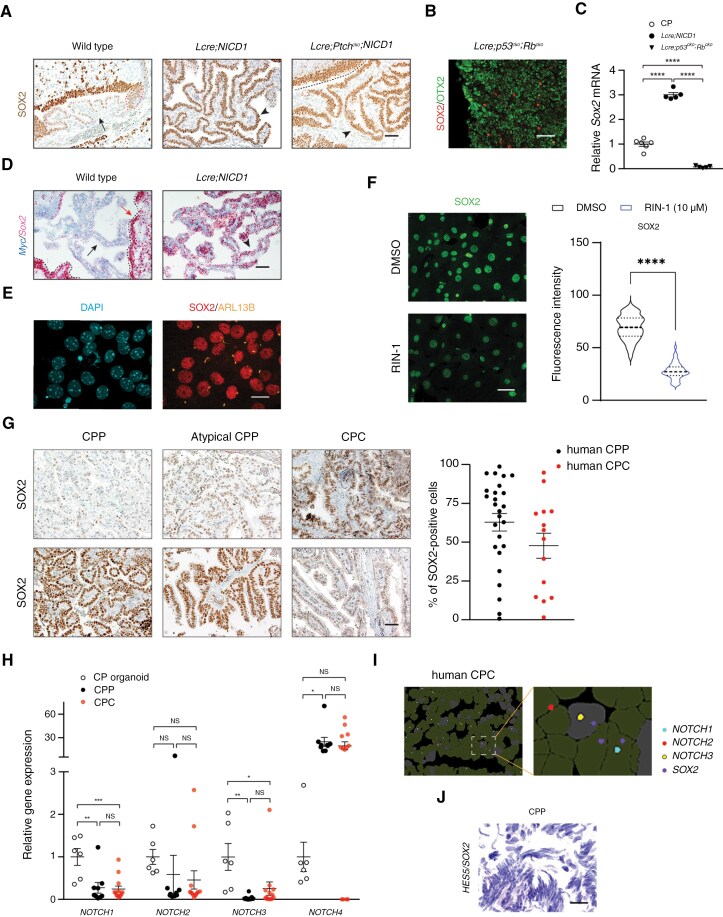
**Increased SOX2 expression in CP tumors in humans and mice.** (A) Immunohistochemistry of SOX2 is shown in the upper rhombic lip/roof plate (red dotted lines) and hindbrain CP (arrow) in wild-type mice, NOTCH-driven CPP and CPC (arrowheads) in *Lcre;NICD1* and *Lcre;Ptch*^*cko*^*;NICD1* animals at embryonic (E) day 14.5 (E14.5). Scale bar, 50 µm. Black dotted line marks the border of the ventricle with SOX2-expressing ependymal cells. (B) Immunofluorescence of SOX2 and OTX2 is shown in *Rb1/Trp53*-deficient CPC in adult *Lcre;p53*^*cko*^*;Rb*^*cko*^ mice. Nuclei are labeled with DAPI. Scale bar, 50 µm. (C) RT-qPCR analysis of *Sox2* expression in wild-type CP, NOTCH-driven CPP, and *Rb1/Trp53*-deficient CPC (*n* = 5 per tissue type, mean ± SEM, 1-way ANOVA, ****P < 0.0001). (D) RNAscope of *Sox2* and *Myc* expression in hindbrain CP (black arrow) in adult wild-type mice, and NOTCH-driven CPP (arrowhead) in adult *Lcre;NICD1* animals. The dotted line marks ventricular walls with ependymal cells (red arrow). Scale bar, 50 µm. (E) Immunofluorescence of SOX2 and ARL13B is shown in NOTCH-driven CPP in adult *Lcre;NICD1* animals. DAPI labels nuclei. Scale bar, 20 µm. (F) Immunofluorescence of SOX2 is shown in NOTCH-driven CP tumor cells treated with DMSO or RIN-1 (10 µM). DAPI labels nuclei. Scale bar, 50 µm. Quantification of fluorescence intensity is shown (*n *= 333 [DMSO]; *n* = 101 [RIN-1], mean ± SEM, 2-tailed unpaired *t* test, ****P < 0.0001). (G) Immunohistochemistry of SOX2 in human CP tumors are shown. Scale bar, 50 µm. (CPC: *n* = 14; CPP and atypical CPP: *n* = 26). All results were obtained from 3 independent experiments. (H) RT-qPCR analysis of gene expression in human CP organoids and CP tumors (*n *= 6 [CP organoid], *n *= 9 [CPP], *n *= 13 [CPC], mean ± SEM, 1-way ANOVA, *P < 0.05; **P < 0.01; ***P < 0.001; NS, nonsignificant). (I) CosMx analysis of the expression of *NOTCH1*, *NOTCH2*, *NOTCH3*, and *SOX2* in a human CPC sample. Boxed region is shown in higher magnification on the right. (J) RNAscope studies of *SOX2* and *HES5* expression in a human CPP. Scale bar, 200 µm.

SOX2 is a critical transcription regulator of progenitor/stem cell functions.^35^ In glioma and medulloblastoma, *Sox2* promotes tumor progression and recurrence.^43,44^ At embryonic (E) day 14.5 (E14.5), robust SOX2 expression was detected in NOTCH-driven CPP, and CPC from *Lmx1a-Cre;Ptch*^*flox/flox*^*;NICD1* (*Lcre;Ptch*^*cko*^*;NICD1*) mice that exhibited constitutive SHH signaling after conditional knockout of *Patched 1* (Figure 2A).^17,33^ In contrast, SOX2 was expressed in a small fraction of tumor cells in *Rb1/Trp53-deficient* CPC (Figure 2B). Postnatally, *Sox2* was markedly upregulated in NOTCH-driven CPP (Figure 2C–E). However, treatment with RIN-1, an inhibitor of the RBPJ subunit of the NOTCH complex,^45^ significantly decreased SOX2 expression in tumor cells (Figure 2F), suggesting that SOX2 expression regulated by NOTCH signaling may contribute to tumor development.

Consistently, SOX2 expression was observed across histological grades in human CP tumor samples that exhibited variations in positive staining area and signal intensity (Figure 2G). RT-qPCR studies revealed the expression of *NOTCH1-3* in human CP tumors, whereas *NOTCH4* expression was significantly increased in CPPs compared to human CP organoids (Figure 2H). CosMx spatial transcriptomics platform uncovered the expression of NOTCH1-3 in ~40% of tumor cells in 1 human CPC sample. Despite relatively low SOX2 expression in this CPC, some tumor cells exhibited co-expression of *SOX2* and *NOTCH* genes (Figure 2I). RNAscope analysis of 3 human CPPs showed co-expression of *SOX2* and NOTCH target *HES5* in a small fraction of tumor cells (Figure 2J). Together, these results suggest an interaction of NOTCH signaling and SOX2 in CP tumors in humans.

### SOX2 Is Essential for the Glial Progenitor-Like Signature and NOTCH-Driven CP Tumor Development

**Figure 3.**
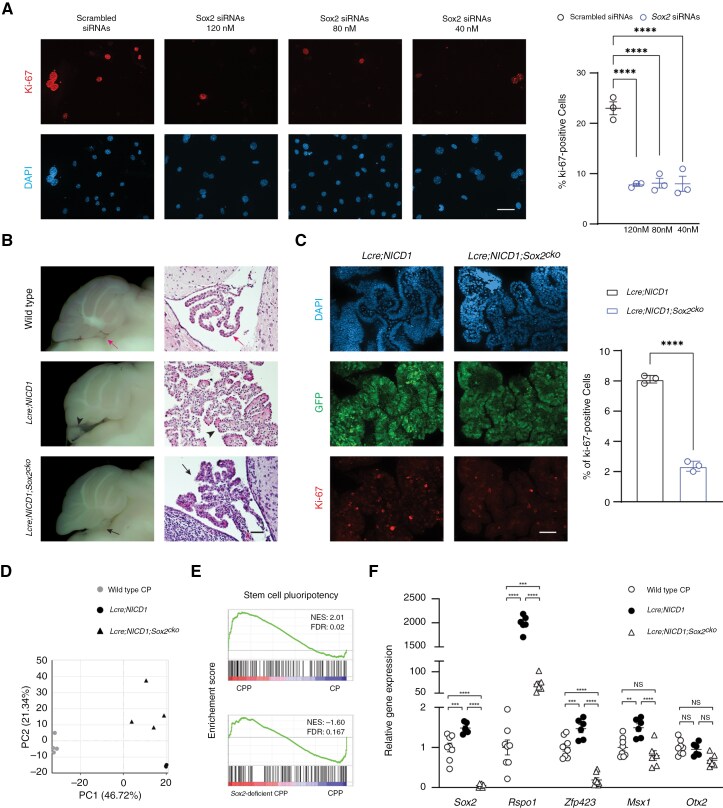
**SOX2 is essential for the glial progenitor-like signature and NOTCH-driven CP tumor development.** (A) Immunofluorescence of Ki-67 is shown in NOTCH-driven CPP cells treated with control scrambled siRNAs or siRNAs against *Sox2* at different concentrations. DAPI labels nuclei. Scale bar, 50 µm. Quantification of Ki-67 expression is shown (*n* = 3 per group, mean ± SEM, 1-way ANOVA, ****P < 0.0001). Data were obtained from 3 independent experiments; see also Figure S2. (B) Bisected brain hemispheres and hematoxylin and eosin (H&E) staining of the hindbrain CP in adult wild-type mice, and CPP in adult *Lcre;NICD1* and *Lcre;NICD1;Sox2*^*cko*^ animals. Red arrows point to wild-type CP, arrowheads point to CPP, black arrows point to SOX2-deficient CPP. Scale bar, 50 µm. (C) Immunofluorescence of Ki-67 and GFP is shown in CPP in *Lcre;NICD1* and *Lcre;NICD1;Sox2*^*cko*^ animals at day E13.5. Nuclei are labeled with DAPI. Scale bar, 50 µm. Quantification of Ki-67 expression is shown (*n* = 3 per group, mean ± SEM, 2-tailed unpaired *t* test, ****P < 0.0001). Results were obtained from 3 independent experiments; also see Figure S3. (D) Principal component analysis (PCA) of CP in wild-type mice, CPP in *Lcre;NICD1* mice, and *Sox2*-deficient CPP in *Lcre;NICD1;Sox2*^*cko*^ animals; also see Figure S3. (E) Gene set enrichment analysis (GSEA) of the effect of *Sox2* loss on NOTCH-driven CP tumors. Pathways regulating pluripotency of the stem cells are shown as an example; also see Figure S3. NES, normalized enrichment score. FDR, false discovery rate. (F) RT-qPCR analysis of gene expression in CP in wild-type mice, and CPP from *Lcre;NICD1* and *Lcre;NICD1;Sox2*^*cko*^ animals (*n *= 8 [CP], *n *= 6 [CPP], mean ± SEM, 1-way ANOVA, **P < 0.01; ***P < 0.001; ****P < 0.0001; NS, nonsignificant); also see Figure S3.

To evaluate the role of *Sox2* in NOTCH-driven CP tumors, knockdown of *Sox2* was performed with siRNAs or a short hairpin RNA (shRNA) against *Sox2* (Figure S2A–S2C). *Sox2* knockdown resulted in significantly reduced Ki-67 expression in tumor cells (Figure 3A, Figure S2D). To delete *Sox2* in tumor cells, a mouse strain carrying conditional alleles of *Sox2* (*Sox2*^*flox/flox*^) was bred with *Lcre;NICD1* mice.^46^*Sox2* loss blunted tumor development and restored cuboidal epithelial morphology in *Lcre;NICD1;Sox2*^*flox/flox*^ (*Lcre;NICD1;Sox2*^*cko*^) animals (Figure 3B, Figure S3A). Consistently, there was markedly reduced Ki-67 expression, indicative of decreased proliferation of *Sox2*-deficient tumor cells in *Lcre;NICD1;Sox2*^*cko*^ mice (Figure 3C). RNA-seq studies were performed to examine the effect of *Sox2* loss on tumor cells (Figure S3B). Principal component analysis revealed distinct gene expression profiles in wild-type CPs, *Sox2*-wild-type and *Sox2*-deficient CPPs (Figure 3D). Gene set enrichment analysis (GSEA) showed enrichment of pathways in regulating stem cell pluripotency in NOTCH-driven CPP using the KEGG database. However, GSEA of gene expression profiles of *Sox2*-deficient CPP resulted in a negative normalized enrichment score for *Sox2*-wild-type CPP, suggesting alterations in these pathways in tumor cells following *Sox2* knockout (Figure 3E). Indeed, the enhanced glial progenitor-like profiles including *Rspo1*, *Zfp423*, and *Msx1* were significantly reduced, whereas *Otx2* mRNA levels remained unaltered in *Sox2*-deficient tumor cells (Figure 3F).

Nonetheless, GSEA showed enrichment of cilium structure and function programs using the GO database (Figure S3C). Consistently, the expression of multiciliation regulators *Gmnc*, *TAp73*, and *Foxj1*, as well as CP epithelial markers *Ttr* and *Aqp1*, were significantly reduced in tumor cells irrespective of *Sox2* loss (Figure S3D). Consequently, tumor cells displayed solitary cilia and lack of AQP1 expression (Figure S3E, S3F), indicating similar defects in multiciliation and epithelial maturation in *Sox2*-deficient tumor cells. Together, these findings indicate that SOX2 maintains the glial progenitor-like signature and promotes NOTCH-driven CP tumor growth.

### SOX2 Transcriptional Targets in NOTCH-Driven CP Tumors Are Enriched in Markers and Regulators of Glial Progenitors in the Rhombic Lip

**Figure 4.**
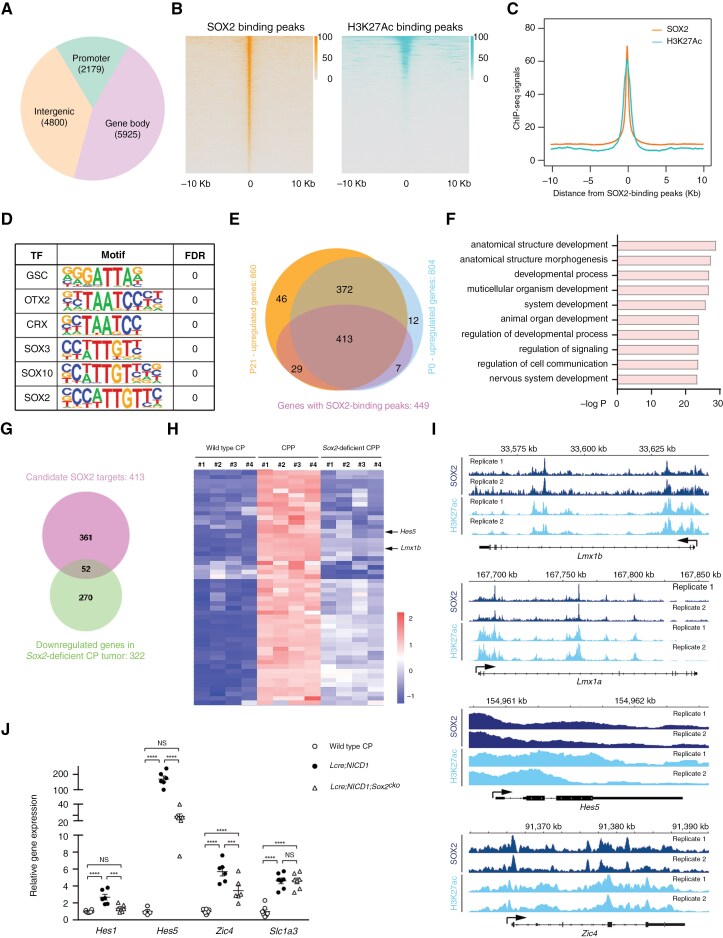
**SOX2 transcriptional targets in CP tumors are enriched in markers and transcriptional regulators of progenitors in the rhombic lip.** (A) Pie chart illustrating the distribution of SOX2-binding sites in relation to genes in NOTCH-driven CPP in *Lcre;NICD1* animals; also see Figure S4. (B) Heatmap of tag densities of SOX2 (left) or H3K23Ac (right) ChIP-seq signals at all of the binding regions identified in ChIP-seq experiments. In each heat map the tag density is plotted for 10 Kb at either side of its binding peak summit. (C) Comparison of SOX2 and H3K27Ac signals generated from ChIP-seq fragment counts in the 20 Kb genomic regions surrounding SOX2 peaks in NOTCH-driven CPP. (D) Logos for the motif enriched in SOX2-binding sequences identified by motif analysis in NOTCH-driven CPP; also see Figure S4. TF: transcription factor; FDR: false discovery rate. (E) Venn diagram shows the overlap of SOX2-associated genes and differentially expressed genes in NOTCH-driven CPPs at days P0 and P21, respectively. (F) GO analysis of candidate SOX2 transcriptional targets in NOTCH-driven CPP. (G) Venn diagram shows the overlap of SOX2 candidate transcriptional targets in (E) and significantly downregulated genes in *Sox2*-deficient tumors. (H) Hierarchical clustering of the expression of 52 candidate SOX2 transcriptional targets identified in (G, FDR < 0.05) in wild-type CP, and *Sox2*-wild-type or *Sox2*-deficient NOTCH-driven CPP. *Lmx1b* and *Hes5* on the heatmap are marked by arrows. (I) The peak density plot of fragment counts is shown in genomic regions that encompass *Lmx1b*, *Lmx1a*, *Hes5*, and *Zic4*, and bound by SOX2 and H3K27Ac, respectively. Genes are labeled in black with sequence in a single exon as a rectangle; also see Figure S4. (J) RT-qPCR analysis of gene expression in CP in wild-type mice, and CPP from *Lcre;NICD1* and *Lcre;NICD1;Sox2*^*cko*^ animals (*n *= 8 [CP], *n *= 6 [CPP], mean ± SEM, 1-way ANOVA, ***P < 0.001; ****P < 0.0001; NS, nonsignificant).

To understand the mechanisms of SOX2 functions in NOTCH-driven CP tumors, ChIP combined with high-throughput sequencing (ChIP-seq) was conducted to characterize SOX2 chromatin binding profile, and that of histone H3 protein with acetylated lysine at N-terminal position 27 (H3K27Ac). Analysis of pooled data uncovered over 10 000 SOX2-bound peaks in promoters/enhancers, gene bodies, and intergenic regions in tumor cells (Figure 4A, B). Consistent with characteristics of a transcription activator, SOX2 binding was frequently associated with H3K27Ac peaks across the genome (Figure 4B, C). Motif analysis showed an enrichment of consensus binding sequence of SOX family of transcription factors in tumor cells (Figure 4D).

We postulated that potential SOX2 transcriptional targets should be elevated in tumor cells. Thus, SOX2-bound genes were compared with upregulated transcripts from RNA-seq data of NOTCH-driven CP tumors, after which 413 potential transcriptional targets were identified (Figure 4E, Table S3). GO analysis revealed an enrichment of pathways in nervous system and brain structure development, as well as signaling regulation (Figure 4F), suggesting a role of SOX2 in the neural progenitor program. The candidate SOX2 targets were further overlapped with downregulated genes identified from RNA-seq data obtained from *Sox2*-deficient CP tumors (Figure 4G). As a result, 52 of the 413 SOX2-bound genes were identified, the expression of which was significantly reduced in *Sox2*-deficient tumor cells (Figure 4G, H). GO analysis reaffirmed enrichment of pathways in brain development (Table S4).

Among these candidate genes, SOX2 binding peaks were present in genomic regions of transcriptional regulators of the hindbrain.^8,9,47^ Specifically, these analyses uncovered LIM homeobox transcription factors *Lmx1a* and *Lmx1b* that displayed SOX2 binding peaks in promoter regions (Figure 4I, Table S3). Both genes were expressed in the glia-like and the epithelial-like compartments of CP tumor (Table S1). RNA-seq data showed that they were upregulated in NOTCH-driven CPP, whereas *Lmx1b* was also downregulated following *Sox2* loss (Tables S2 and S3, Figure 4G, H). Moreover, SOX2 binding was also detected in the promoters of *Zic1*-*Zic4*, NOTCH targets *Hes5* and *Hes1*, as well as 8 glial progenitor markers in NOTCH-driven CP tumors (Figures 1B, D, I, and 4I, Figure S4A, Tables S1 and S3). Their expression was upregulated in NOTCH-driven CPP, but decreased in *Sox2*-deficient tumor cells (Tables S2 and S3, Figure 4J).

Notably, SOX2-bound sequences were enriched in OTX2 motif (Figure 4D). To address this issue, ChIP-seq studies were performed to identify OTX2 binding profile in NTOCH-driven CP tumors. An extensive overlap was observed between SOX2-bound and OTX2-bound genomic fragments (Figure S4B–S4D). GO analysis showed that genes with enhanced OTX2 binding were enriched in pathways regulating brain development (Figure S4E). Co-occupancy of OTX2 was detected in the promoters of markers and transcriptional regulators of glial progenitors in the rhombic lip, including *Lmx1b*, *Lmx1a*, *Zic3*, *Zic4*, *Hes5*, *Hes1*, and *Slc1a3* (Figure S4F). Consistently, co-immunoprecipitation revealed association of SOX2 and OTX2 in NOTCH-driven CP tumors (Figure S4G). Together, these results suggest that SOX2 may collaborate with OTX2 in regulating progenitor/stem cell-like identity in NOTCH-driven CP tumors and hindbrain development.

### SOX2 Regulates LMX1A and LMX1B Expression in NOTCH-Driven CP Tumors

**Figure 5.**
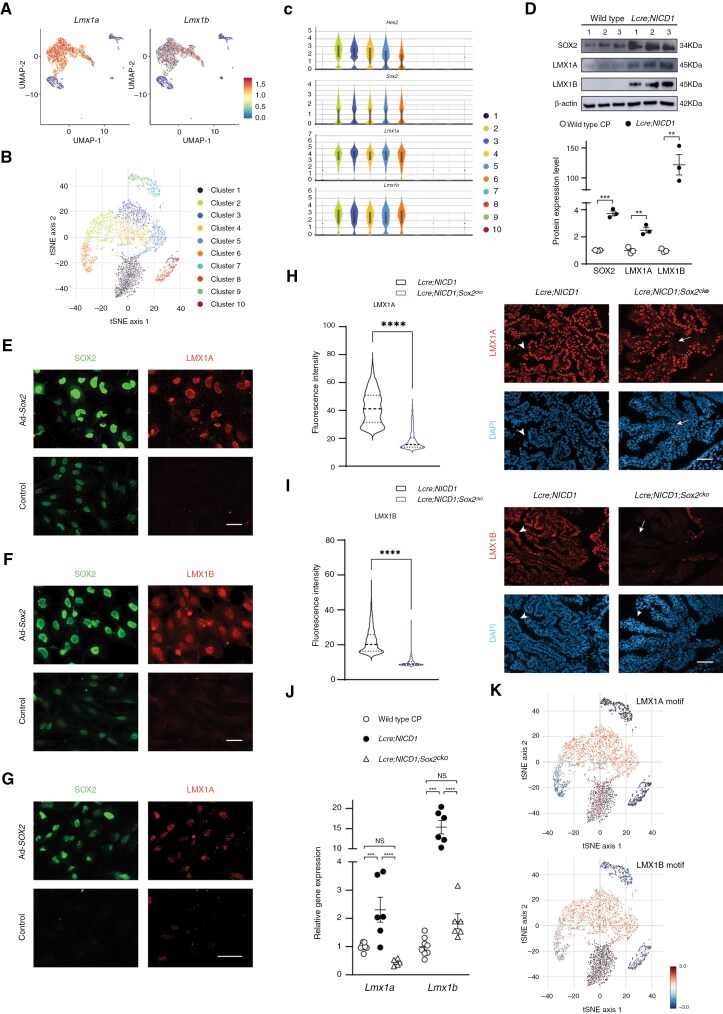
**SOX2 regulates transcription factors LMX1A and LMX1B in NOTCH-driven CP tumors.** (A) UMAP of 6428 single-nucleus profiles from a NOTCH-driven CPP colored by *Lmx1a* and *Lmx1b* expression, respectively. (B) t-distributed stochastic neighbor embedding (t-SNE) plot shows the annotated scATAC-seq profiles of different cell populations in NOTCH-driven CPP. Different subclusters of cells are marked by different colors. (C) Violin plots show the activity of different genes in each subcluster of cells. (D) Western blot analysis of the expression of SOX2, LMX1A, and LMX1B in CP in wild-type mice, and NOTCH-driven CPP in *Lcre;NICD1* animals (*n* = 3 per group, mean ± SEM, 2-tailed unpaired *t* test, **P < 0.01; ***P < 0.001). Three independent experiments were conducted; also see Figure S5. (E, F) Immunofluorescence of LMX1A (E) and LMX1B (F) is shown in *Rb1/Trp53*-deficient CPC cells infected with viruses expressing SOX2. SOX2 labels infected cells. Scale bars, 50 µm. Three independent experiments were conducted. (G) Immunofluorescence of LMX1A is shown in *TP53*-deficient human CPC cells infected with viruses expressing SOX2. SOX2 labels infected cells. Scale bar, 50 µm. Three independent experiments were conducted. (H, I) Immunofluorescence of LMX1A (H) LMX1B (I) is shown in CPP in *Lcre;NICD1* and *Lcre;NICD1;Sox2*^*cko*^ animals at postnatal (P) day 7 (P7). Arrowheads point to tumor cells, arrows point to SOX2-deficient tumor cells. DAPI labels nuclei. Scale bars, 50 µm. Fluorescence intensity is quantified (*n *= 1101 [LMX1A], *n *= 754 [LMX1B] for NOTCH-driven CPP cells; *n* = 457 [LMX1A], *n* = 563 [LMX1B] for *Sox2*-deficient tumor cells; mean ± SEM, 2-tailed unpaired *t* test, ****P < 0.0001). The experiments were repeated 3 times independently; also see Figures S6 and S7. (J) RT-qPCR analysis of the expression of *Lmx1a* and *Lmx1b* in CP in wild-type mice, and CPP from *Lcre;NICD1* and *Lcre;NICD1;Sox2*^*cko*^ animals (*n *= 8 [CP], *n *= 6 [CPP], mean ± SEM, 1-way ANOVA, ***P < 0.001; ****P < 0.0001; NS, nonsignificant). Results were obtained from 1 experiment. (K) t-SNE plots show that motifs of LMX1A and LMX1B are enriched in tumor cell subclusters in NOTCH-driven CPP. Colors represent average gene activity score of cells in each subcluster. Dark red means high gene activity score, blue means low gene activity score.

The identification of potential SOX2 transcriptional targets prompted us to characterize the expression of LIM homeobox genes in NOTCH-driven CP tumors. UMAP analysis of single-nucleus profiles revealed enrichment of the expression of *Lmx1a* and *Lmx1b* in the epithelial-like and the glia-like cell compartments (Figure 5A, Table S1). ScATAC-seq studies identified multiple cell groups in NOTCH-driven CP tumors using differential accessible peaks (Figure 5B). Tumor cell groups were assigned based on chromatin accessibility of genes expressed in tumor cells, including *Hes5* and *Sox2*, whereas regulatory regions of *Lmx1a* and *Lmx1b* were accessible only in tumor cell groups (Figure 5C). Consistently, RT-qPCR and RNA-seq studies using tumor bulk showed significantly increased *Lmx1a* and *Lmx1b* expression, along with increased *Sox2* levels, compared to wild-type CP from birth to adult stage (Figure S5A, S5B, Table S2). At protein level, the expression of LMX1A, LMX1B, and SOX2 was markedly elevated in tumor cells (Figure 5D). At the cellular level, immunofluorescence revealed nuclear expression of LMX1A, LMX1B, and SOX2 in NOTCH-driven CPP and CPC, respectively (Figure S5C–S5F).

To explore the interaction of SOX2 with LMX1 transcription factors, murine *Rb1/Trp53*-deficient CPC cells and a human CPC cell line were infected with viruses expressing SOX2, respectively.^17^ Immunofluorescence showed that SOX2 overexpression increased the expression of LMX1A and LMX1B in infected tumor cells (Figure 5E–G). Conversely, *Sox2* loss resulted in significantly reduced LMX1A expression, whereas LMX1B was lost in *Sox2*-deficient tumor cells after birth (Figure 5H–J, Figure S6A, S6B). In agreement, knockdown of *Sox2* markedly decreased LMX1A and LMX1B expression in NOTCH-driven CP tumor cells (Figure S6C–S6E). Consistently, analysis of accessible genomic fragments from scATAC-seq data revealed enrichment of LMX1A and LMX1B binding motif in tumor cells (Figure 5K). Together, these results indicate that SOX2 regulates the expression of LMX1A and LMX1B to contribute to the epigenetic landscape of NOTCH-driven CP tumors.

### SOX2 Regulates LMX1A and LMX1B Expression in the Rhombic Lip/CP During Development

SOX2-LMX1 signaling was further investigated during embryogenesis.^7–9^ First, strong expression of LMX1A, LMX1B, and SOX2 was detected in NOTCH-driven CP tumor cells at day E13.5 (Figure S7A, S7B). After loss of *Sox2*, LMX1A and Ki-67 expression was greatly reduced, whereas the expression of LMX1B was abolished in *Sox2*-deficient CP tumor cells from *Lcre;NICD1;Sox2*^*cko*^ embryos (Figure S7A–S7C). At day E13.5, SOX2 expression was detected in the rhombic lip, but was gradually downregulated in CP epithelial cells (Figure S8A). The expression of LMX1A and OTX2 were initiated in the rhombic lip and upregulated in CP epithelial cells, whereas LMX1B was detected in the rhombic lip but became downregulated in epithelial cells (Figure S8A, S8B). In *Lcre;Sox2*^*cko*^ embryos, LMX1A and LMX1B expression in the rhombic lip and CP epithelium were markedly attenuated or completely lost, while the expression of OTX2 remained unaffected by SOX2 loss (Figure S8A–S8C). Taken together, these results indicate that LMX1A and LMX1B act in a SOX2-dependent manner during CP development.

### LMX1A and LMX1B Reconstitute SOX2 Functions to Support Tumor Cell Proliferation

**Figure 6.**
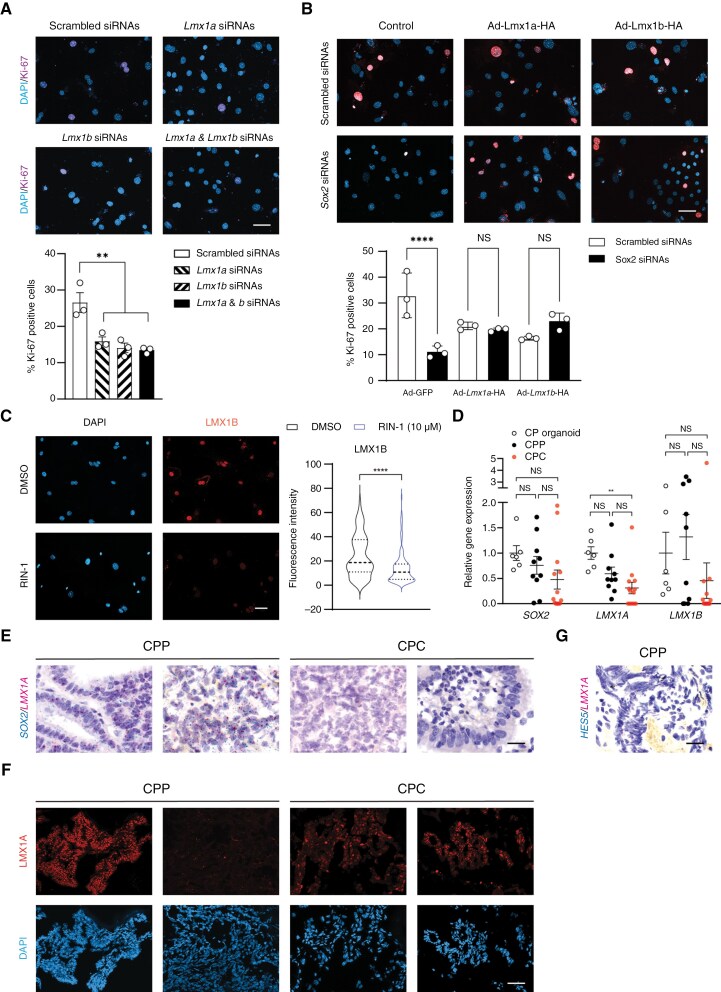
**LMX1A and LMX1B mediate SOX2 functions to support tumor cell proliferation.** (A) Immunofluorescence of Ki-67 is shown in NOTCH-driven CPP treated with control scrambled siRNAs or siRNAs against *Lmx1a* and/or *Lmx1b* (40 nM). DAPI labels nuclei. Scale bar, 50 µm. Quantification of Ki-67 expression is shown (*n* = 3 per group, mean ± SEM, 1-way ANOVA, **P < 0.01). The experiments were repeated 3 times independently; also see Figure S9. (B) Immunofluorescence of Ki-67 is shown in NOTCH-driven CPP treated with control scrambled siRNAs or siRNAs against *Sox2* (40 nM), and infected with viruses expressing HA-tagged *Lmx1a*, *Lmx1b*, or control viruses. DAPI labels nuclei. Scale bar, 50 µm. Quantification of Ki-67 expression is shown (*n* = 3 per group, mean ± SEM, 1-way ANOVA, ****P < 0.0001; NS, nonsignificant). Three independent experiments were conducted; also see Figure S9. (C) Immunofluorescence of LMX1B is shown in NOTCH-driven CPP cells treated with DMSO or RIN-1 (10 µM). DAPI labels nuclei. Scale bar, 50 µm. Quantification of fluorescence intensity is shown (*n *= 233 [DMSO]; *n* = 201 [RIN-1], mean ± SEM, 2-tailed unpaired *t* test, ****P < 0.0001). (D) RT-qPCR analysis of the expression of *SOX2*, *LMX1A*, and *LMX1B* in human CP organoids and CP tumors (*n *= 6 [CP organoid], *n* = 9 [CPP], *n* = 13 [CPC]; mean ± SEM, NS, nonsignificant). The experiments were repeated 1 time independently. (E) RNAscope studies of *LMX1A* and *SOX2* expression in human CP tumors. Scale bar, 200 µm. (F) Immunofluorescence of LMX1A in human CP tumor samples is shown. DAPI labels nuclei. Scale bar, 50 µm. Three independent experiments were conducted. (G) RNAscope studies of *LMX1A* and *HES5* expression in a human CPP. Scale bar, 200 µm.

To assess the role of LMX1A and LMX1B in tumorigenesis, NOTCH-driven CP tumor cells were isolated from *Lcre;NICD1* mice and treated with siRNAs against *Lmx1a* and/or *Lmx1b*, and control scrambled siRNAs (Figure S9A). Knockdown of *Lmx1a* and/or *Lmx1b* dramatically decreased Ki-67 expression in tumor cells (Figure 6A), indicating that LMX1A and LMX1B are required for tumor cell proliferation. To determine whether SOX2 supports tumor cell proliferation through regulating LMX1 gene expression, primary tumor cells from *Lcre;NICD1* mice were infected with viruses expressing HA-tagged LMX1A or LMX1B, or control viruses, followed by treatment with siRNAs against *Sox2* or control siRNAs for 72 h (Figure S8B, S8C). Immunofluorescence demonstrated that Ki-67 expression was reduced after *Sox2* knockdown in tumor cells infected with control viruses, indicative of decreased tumor cell proliferation (Figure 6B). However, *Sox2* knockdown failed to reduce Ki-67 expression in tumor cells with virus-mediated overexpression of LMX1A or LMX1B (Figure 6B), suggesting that LMX1 transcription factors support NOTCH-driven CP tumor cell proliferation in the absence of SOX2. To determine whether LMX1 gene expression can be targeted by NOTCH inhibition, tumor cells were treated with RIN-1. Immunofluorescence revealed a significant decrease in LMX1B expression in tumor cells following RIN-1 treatment (Figure 6C). Together, these results indicate that LMX1A and LMX1B act downstream of SOX2 to support tumor cell proliferation, whereas NOTCH inhibition suppresses SOX2-LMX1 signaling in CP tumor cells.

To evaluate SOX2 and LMX1 signaling in human diseases, RT-qPCR studies were conducted that revealed extensive *LMX1A* and *LMX1B* expression, along with variable *SOX2* mRNA levels, in human CP tumors (Figure 6D). Moreover, RNAscope studies were conducted in 9 human CP tumors (3 samples for each grade). Results from these studies uncovered extensive SOX2 and LMX1A expression in all grade 1 and 2 CP tumors examined, whereas all 3 grade 3 tumors exhibited low levels of SOX2 and LMX1A expression (Figure 6E). Consistently, immunofluorescence also showed that LMX1A was expressed in a subset of CP tumor in humans (Figure 6F). RNAscope studies of 3 human CP tumors (1 grade 1 tumor, and 2 grade 2 tumors) revealed co-expression of *HES5* and *LMX1A* in a fraction of tumor cells (Figure 6G). Together, these results suggest an important role of SOX2-LMX1 signaling in CP tumors in humans.

## Discussion

CP tumors are rare brain tumors that most commonly arise in young children. Despite its dismal prognosis, standards for the treatment of CPC have not been established.^22,23^ The development of safer and more effective therapies for CPC requires a better understanding of its biology. Though CP tumors lack recurrent driver alterations except for mutations of *TP53*, analysis of transcriptional and epigenetic characteristics in CPC allowed the development of experimental models to study these malignancies.^17,30–33^ Knowledge of the molecular factors driving CP tumors may assist in risk and treatment stratification, and patient management in future clinical trials.

In the current study, single-nucleus transcriptomics and single-cell epigenetics technologies were utilized to decipher cellular, epigenetic, and molecular features of NOTCH-driven CP tumor in an animal model. SnRNA-seq and scATAC-seq data revealed cell clusters in tumor samples similar to those in normal CP, including endothelial, immune, mesenchymal, neuronal, epithelial-like, and glia-like populations. The cellular diversity within the tumor microenvironment suggests interactions among different cell groups in tumor development. A molecular, cellular, and spatial transcriptomics map of the CP across different ages and anatomical locations represents a framework to achieve better understanding of these interactions. It was shown recently that a binary glial progenitor pool in the rhombic lip gives rise to the neuronal population and epithelial cells in the CP.^3^ Consistently, analysis of single-nucleus profiles from CP tumors revealed cell groups that resemble the glial progenitor and the epithelial cell groups in the CP (named glia-like and epithelial-like, respectively). Tumor cells in the epithelial-like cell cluster exhibited active NOTCH signaling and adopted progenitor-like characteristics of the glia-like cell compartment. Further analysis of the epithelial-like compartment generated multiple subgroups that mostly retained the glial progenitor-like features. Cell trajectory analysis juxtaposed the glia-like compartment upstream of these epithelial-like subgroups that dynamically navigate distinct differentiation states, indicating that NOTCH-driven CP tumors arise from the bipotential glial progenitor pool in the rhombic lip, and undergo differentiation and expansion in the epithelial-like compartment. In agreement, previous studies showed that NOTCH signaling suppresses multiciliation of progenitors, whereas constitutive SHH signaling leads to the expansion of upper rhombic lip and collaborates with NOTCH signaling to drive CPC formation.^17,33^

The glial progenitor-like profile in NOTCH-driven CP tumor is characterized by enhanced expression of *Rspo1*, *Zfp423*, *Gdf7*, *Msx1*, *Zic3/4*, and *Sox2*, genes normally expressed in the rhombic lip progenitor pool capable of neuronal or epithelial differentiation during hindbrain development.^3,6–11^ Their expression and those of other glial progenitor markers were maintained as tumor cells transition through multiple differentiation states, suggesting an important role of the progenitor-like program in CP tumorigenesis. However, the molecular mechanisms underlying the progenitor-like signature remain unclear. NOTCH signaling maintains neural stem cells in developing and adult brains, and contributes to SOX2 regulation during neural stem cell induction.^48^ In medulloblastoma and glioma, SOX2 maintains stem cell identity and promotes tumor progression and recurrence.^43,44^ A recent report showed that SOX2 expression is correlated with high-grade CP tumors.^36^ In this study, our results demonstrate that SOX2 acts downstream of NOTCH signaling to maintain the expression of genes associated with glial progenitor-like characteristics, and plays a crucial role to support the proliferation and growth of NOTCH-driven CP tumors. In agreement with previous studies, active NOTCH signaling was detected in a subset of CP tumors in humans, whereas analysis of high-resolution spatial transcriptomics data suggests that SOX2 may act downstream of NOTCH signaling in human CP tumors.

Integrative ChIP-seq and RNA-seq studies identified putative SOX2 transcriptional target genes in tumor cells, including markers of progenitors in the rhombic lip, transcriptional regulators of hindbrain development such as *Zic1-Zic4*, NOTCH targets *Hes5* and *Hes1*, and LMX1A and LMX1B transcription factors that play a crucial role in the development of the rhombic lip and the CP.^9,11,47,49^ SnRNA-seq and scATAC-seq data confirmed their activities in NOTCH-driven CP tumors. Thus, SOX2 depends on diverse mechanisms to regulate progenitor program activities in NOTCH-driven CP tumors, including regulation of NOTCH pathway targets *Hes5* and *Hes1*. Interestingly, motif analysis suggested close proximity of SOX2 and OTX2 in promoters, whereas comparison of the chromatin binding profiles SOX2 and OTX2 revealed an extensive overlap of genes bound to SOX2 and OTX2. In agreement, co-immunoprecipitation studies demonstrated the association of SOX2 and OTX2 in CP tumor cells. Therefore, SOX2 may regulate transcription either through direct binding to regulatory regions, or through complexing with OTX2 and/or other partners in binding to promoters of transcriptional targets in NOTCH-driven CP tumors.

In NOTCH-driven CP tumors, gene expression studies demonstrated SOX2-dependent expression of LMX1A and LMX1B in both the epithelial-like and the glia-like cell compartments: LMX1B activity critically depends on SOX2, whereas LMX1A is partially dependent on SOX2 expression. Both genes are necessary and sufficient to support SOX2 functions in tumor cell proliferation, and thus comprise an important part of the SOX2-dependent progenitor-like program in tumor development. In parallel, SOX2 plays an important role in the specification of glial progenitors in the rhombic lip by regulating LMX1A and LMX1B expression. Though LMX1B expression in the rhombic lip is abrogated in the absence of SOX2, LMX1A expression is significantly decreased after SOX2 deletion. LMX1B activity is dispensable to CP development, whereas LMX1A plays a more prominent role in the rhombic lip and CP development.^9,11^ Therefore, these results demonstrate that SOX2-LMX1 signaling functions as a critical regulator of progenitor/stem cell-like identity in CP development and tumorigenesis. Consistently, previous studies showed that LMX1A-positive progenitors in the rhombic lip give rise to NOTCH-driven CP tumors.^17,33^

Importantly, this study uncovered co-expression of SOX2 and LMX1A in all human CPP samples examined. Moreover, SOX2 overexpression activated LMX1A expression in a human CPC cell line. The aberrant SOX2 and LMX1A expression in human CP tumors suggests that SOX2-LMX1 signaling plays an important role in tumor development, and may serve as a therapeutic target.^50^ Consistently, our results demonstrated that inhibition of NOTCH signaling suppresses SOX2-LMX1 signaling in tumor cells. The abnormal NOTCH activity in a subset of CP tumors in humans supports NOTCH inhibition as a potential treatment strategy. Indeed, treatment with a NOTCH inhibitor has been previously shown to promote multiciliation and decrease CP tumor growth in mice.^17^ This study may shed light on other brain tumors characterized by a progenitor-like signature. The limitations of this study lie in the need for more human tumor and xenograft studies to validate these conclusions. A detailed understanding of LMX1A/B regulation and downstream signaling will be critical for developing strategies to target this pathway in CPC.

## Supplementary Material

noaf085_suppl_Supplementary_Figures

noaf085_suppl_Supplementary_Table_S1

noaf085_suppl_Supplementary_Table_S2

noaf085_suppl_Supplementary_Table_S3

noaf085_suppl_Supplementary_Table_S4

## Data Availability

Data generated or analyzed for this study will be made available upon reasonable request.
