# Supplementary material for: SOX2 commands LIM homeobox transcription factors in choroid plexus development and tumorigenesis
Source: Neuro Oncol. 2025 Mar 25;27(8):2006–22. doi: 10.1093/neuonc/noaf085 (PMC12448815; doi:10.1093/neuonc/noaf085)
Supplement: noaf085_suppl_Supplementary_Figures [file noaf085_suppl_supplementary_figures.docx]

**
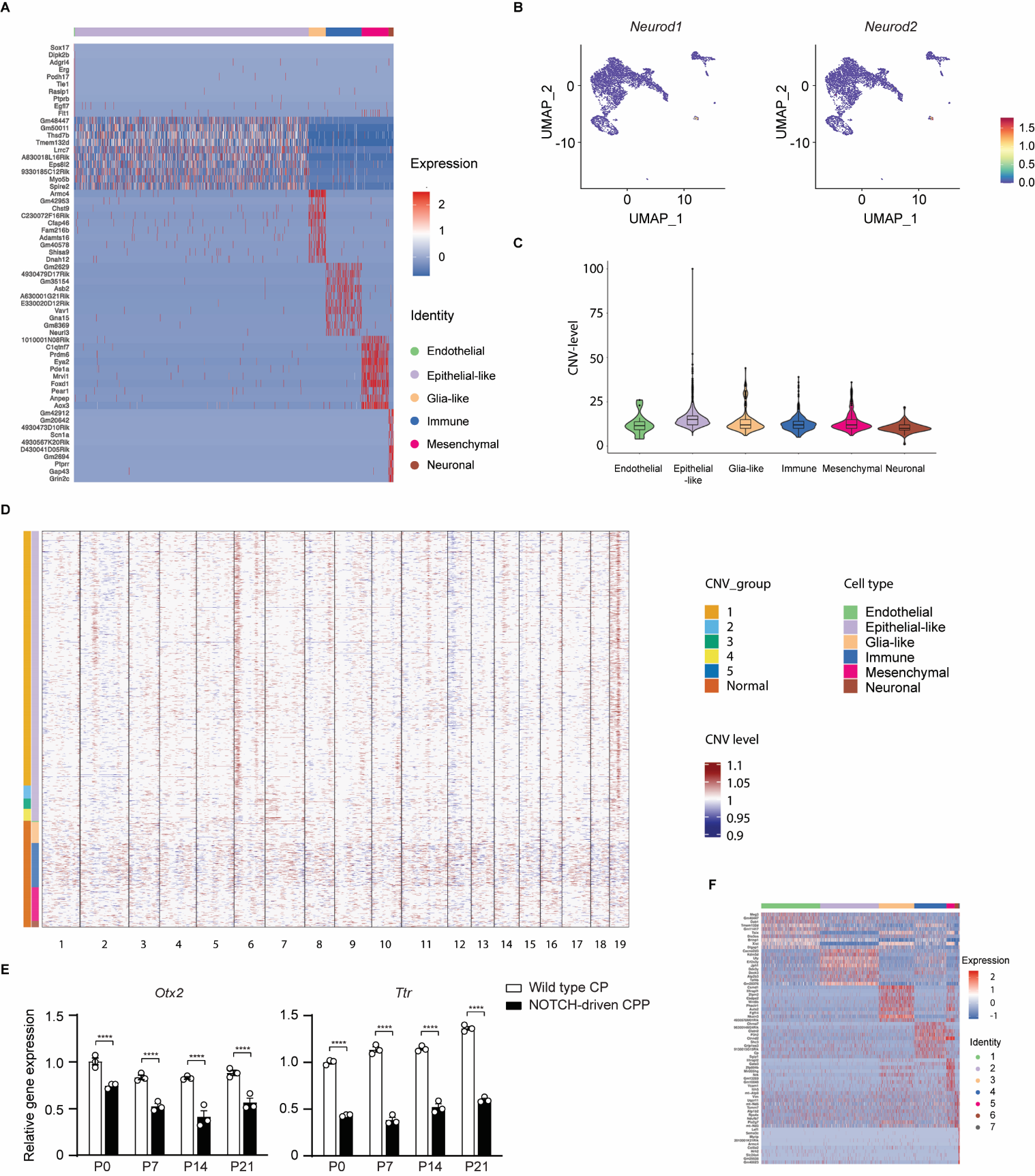
**

**Figure S1. SnRNA-seq reveals cellular diversity and dynamic molecular signature of NOTCH-driven CPP, related to Figure 1.** (A) Heatmap of the expression top 10 genes in each cell population identified from 6,428 single-nucleus profiles of a NOTCH-driven CPP from an adult *Lcre;NICD1* mouse. (B) The expression of neuronal markers in NOTCH-driven CPP. UMAP of 6,428 single-nucleus profiles from snRNA-seq in NOTCH-driven CPP, colored by expression of *Neurod1* and *Neurod2*. (C) Violin plots of copy number variation (CNV) estimation in cell populations identified by snRNA-seq in NOTCH-driven CPP. (D) Heatmap representations of copy number profiles of each chromosome in cell populations identified by snRNA-seq in NOTCH-driven CPP. (E) RT-qPCR analysis of *Otx2* and *Ttr* mRNA levels in NOTCH-driven CPP and wild type CP (*n* = 3 per time point per genotype, mean ± s.e.m., two-tailed unpaired *t*-test, *****P* < 0.0001). Three independent experiments were conducted. (F) Heatmap of gene expression of seven distinct groups within the epithelial-like tumor cell compartment in NOTCH-driven CPP.

**
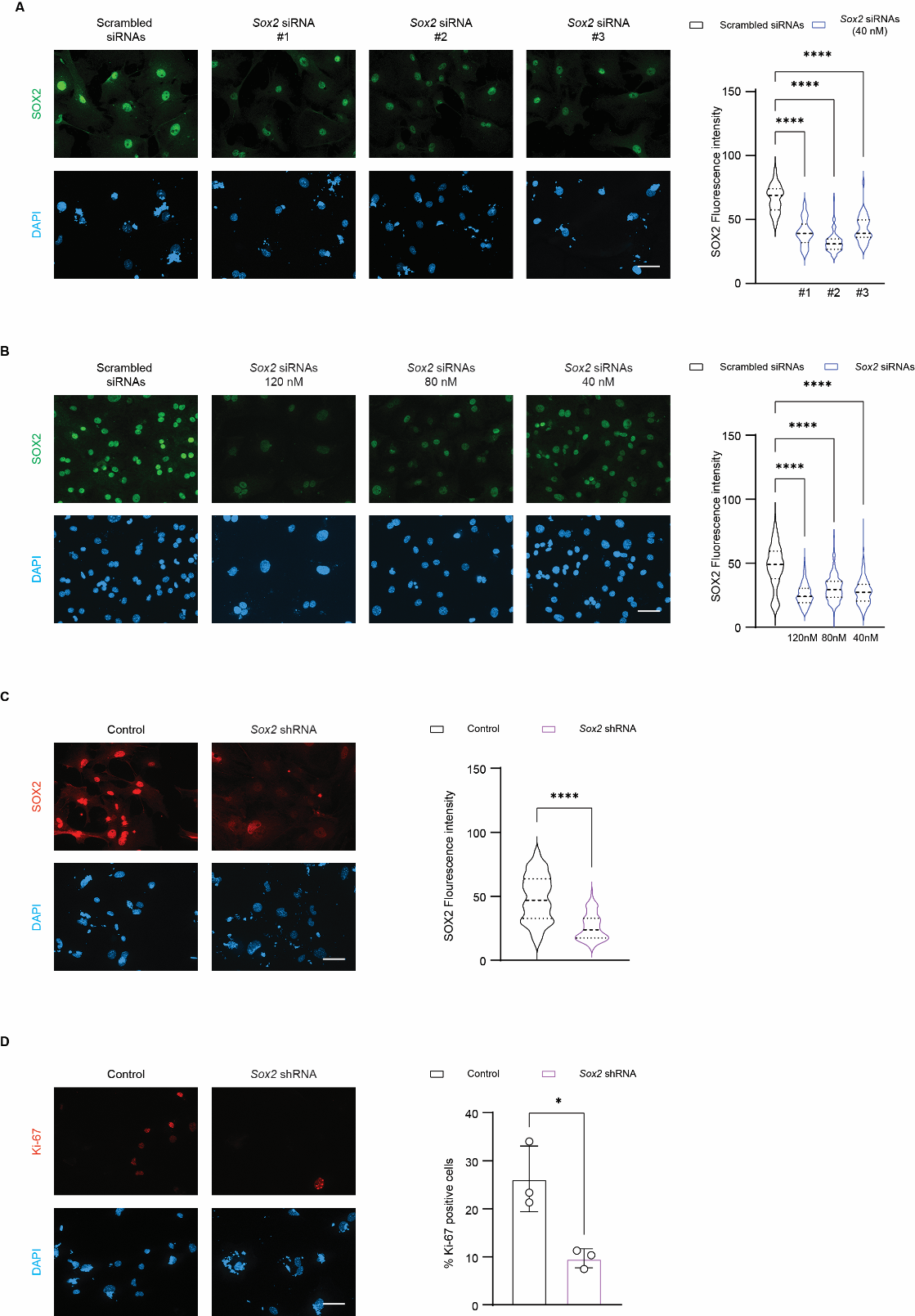
**

**Figure S2. *Sox2* knockdown in NOTCH-driven CPP, related to Figure 3.** (A, B) Immunofluorescence of SOX2 (green) is shown in NOTCH-driven CPP treated with control scrambled siRNAs, individual siRNAs [40 nM] (A), or pools of siRNAs against *Sox2* at different concentrations (B). DAPI (blue) labels nuclei. Scale bars, 50µm. SOX2 fluorescence intensity is quantified (*n* = 50 cells per group [A]; *n =* 583 cells [120 nM], *n =* 563 cells [80 nM], *n =* 653 cells [40 nM], mean ± s.e.m., one-way ANOVA, *****P* < 0.0001). Three independent experiments were conducted. (C) Immunofluorescence of SOX2 (red) is shown in NOTCH-driven CPP infected with control viruses or viruses expressing shRNAs against *Sox2*. DAPI (blue) labels nuclei. Scale bar, 50µm. SOX2 fluorescence intensity is quantified (mean ± s.e.m., two-tailed unpaired *t*-test, *****P* < 0.0001). Three independent experiments were conducted. (D) Immunofluorescence of Ki-67 (red) is shown in NOTCH-driven CPP infected with control viruses, or viruses expressing shRNAs against *Sox2*. DAPI (blue) labels nuclei. Scale bar, 50µm. Quantification of Ki-67 expression is shown (*n =* 3, mean ± s.e.m., two-tailed unpaired *t*-test, **P* < 0.05). Results were obtained from three independent experiments.

**
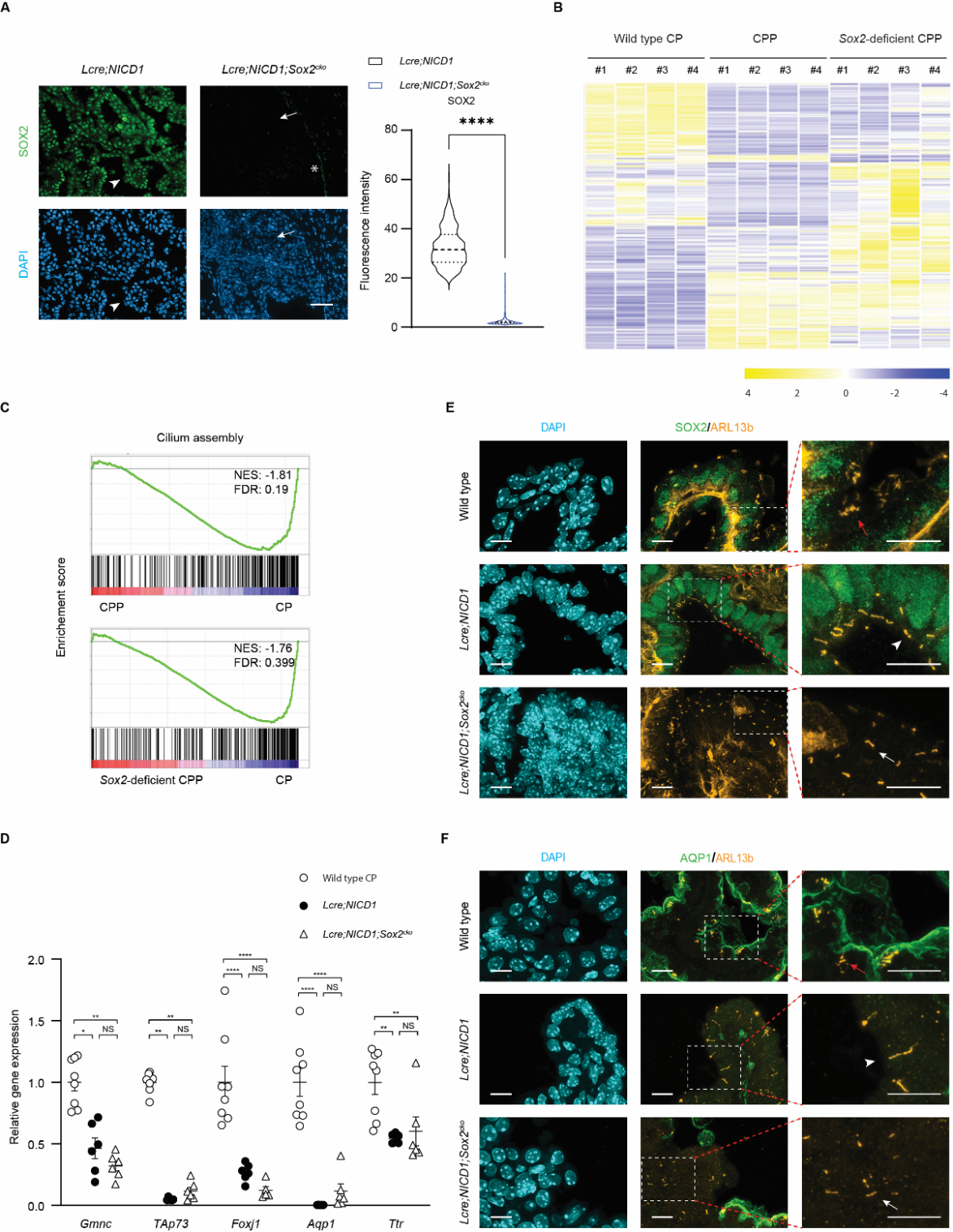
**

**Figure S3. Multiciliation defects in NOTCH-driven CPP remains unaltered by *Sox2* loss, related to Figure 3.** (A) Immunofluorescence of SOX2 (green) is shown in CPP in *Lcre;NICD1* and *Lcre;NICD1;Sox2^cko^* animals at postnatal (P) day 18 (P18). Arrowheads point to tumor cells, arrows point to SOX2-deficient tumor cells, and white asterisk marks SOX2-expressing ependymal cells. DAPI (blue) labels nuclei. Scale bar, 50µm. SOX2 fluorescence intensity is quantified (*n* = 463 for CPP, *n* = 195 for SOX2-deficient tumor cells; mean ± s.e.m., two-tailed unpaired *t*-test, *****P* < 0.0001). Three independent experiments were conducted. (B) Non-hierarchical clustering of differentially expressed genes between CP in wild type mice, CPP in *Lcre;NICD1* mice, and *Sox2*-deficient CPP in *Lcre;NICD1;Sox2^cko^* animals (n = 4; one-way ANOVA, FDR < 0.05). (C) GSEA of CPP in *Lcre;NICD1* mice, *Sox2*-deficient CPP in *Lcre;NICD1;Sox2^cko^* animals, and CP in wild type mice. Cilium assembly program was shown as an example. (D) RT-qPCR analysis of gene expression in the CP in wild type mice, and CPP from *Lcre;NICD1* and *Lcre;NICD1;Sox2^cko^* animals (*n* = 8 [CP], *n* = 6 [CPP]; mean ± s.e.m., one-way ANOVA, **P* < 0.05; ***P* < 0.05; *****P* < 0.0001; NS, non-significant). Three independent experiments were conducted. (E, F) Immunofluorescence of SOX2 (E, green), AQP1 (F, green) and ARL13B (yellow) is shown in CPP in *Lcre;NICD1* and *Lcre;NICD1;Sox2^cko^* animals, respectively. Red arrows point to wild type CP epithelial cells, arrowheads point to SOX2-wild type tumor cells in in *Lcre;NICD1* mice, and white arrows point to SOX2-deficient tumor cells *Lcre;NICD1;Sox2^cko^* mice. Region in bracket is shown in higher magnification on the right. DAPI (blue) labels nuclei. Scale bars, 20µm. Results were obtained from three independent experiments.

**
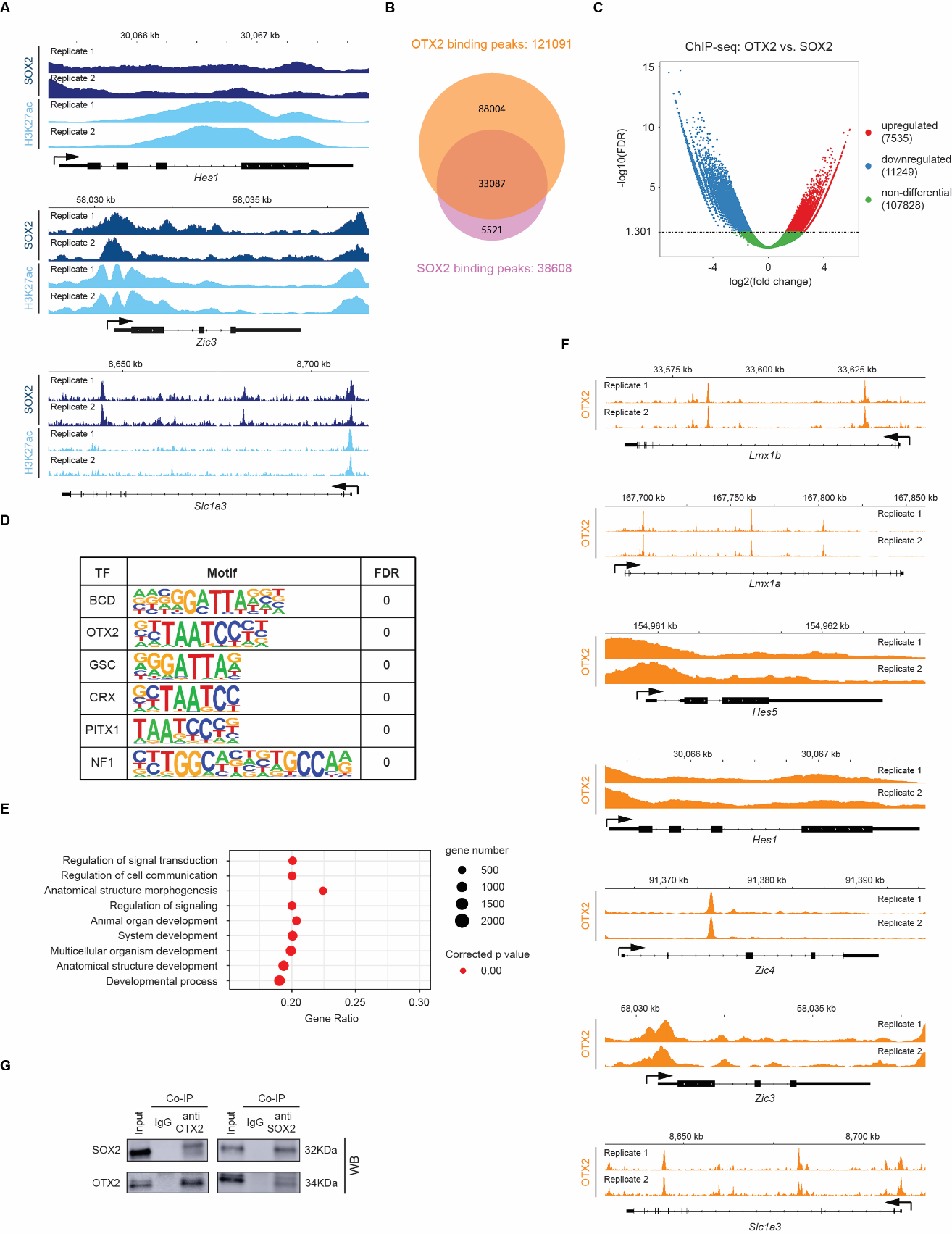
**

**Figure S4.** **Collaboration of OTX2 and SOX2 in transcriptional regulation in CP tumors, related to Figure 4.** (A) Peak density plot of fragment counts is shown in genomic regions that encompass *Hes1*, *Zic3*, and *Slc1a3* and bound by SOX2 and H3K27Ac, respectively. Genes are labeled in black with sequence in a single exon as a rectangle. (B) Venn diagram shows the overlap of SOX2-binding fragments with OTX2-binding fragments in NOTCH-driven CPP in *Lcre;NICD1* animals. (C) Volcano plot shows the differences in tag densities of OTX2-bound and SOX2-bound in ChIP-seq signals at all of the binding regions identified in ChIP-seq experiments. (D) Logos for the motif enriched in OTX2-binding sequences in NOTCH-driven CPP. TF: transcription factor; FDR: false discovery rate. (E) GO analysis of upregulated OTX2-binding sequences compared to SOX2-binding sequences in NOTCH-driven CPP. (F) Peak density plot of fragment counts is shown in *Lmx1b*, *Lmx1a*, *Hes5*, *Hes1*, *Zic4*, *Zic3*, and *Slc1a3* genomic regions bound by OTX2 and H3K27Ac, respectively. Genes are labeled in black with sequence in a single exon as a rectangle. (G) Co-IP using NOTCH-driven CPP with anti-OTX2, and anti-SOX2 antibodies, respectively. Western blot was performed with anti-OTX2 and anti-SOX2 antibodies.

**
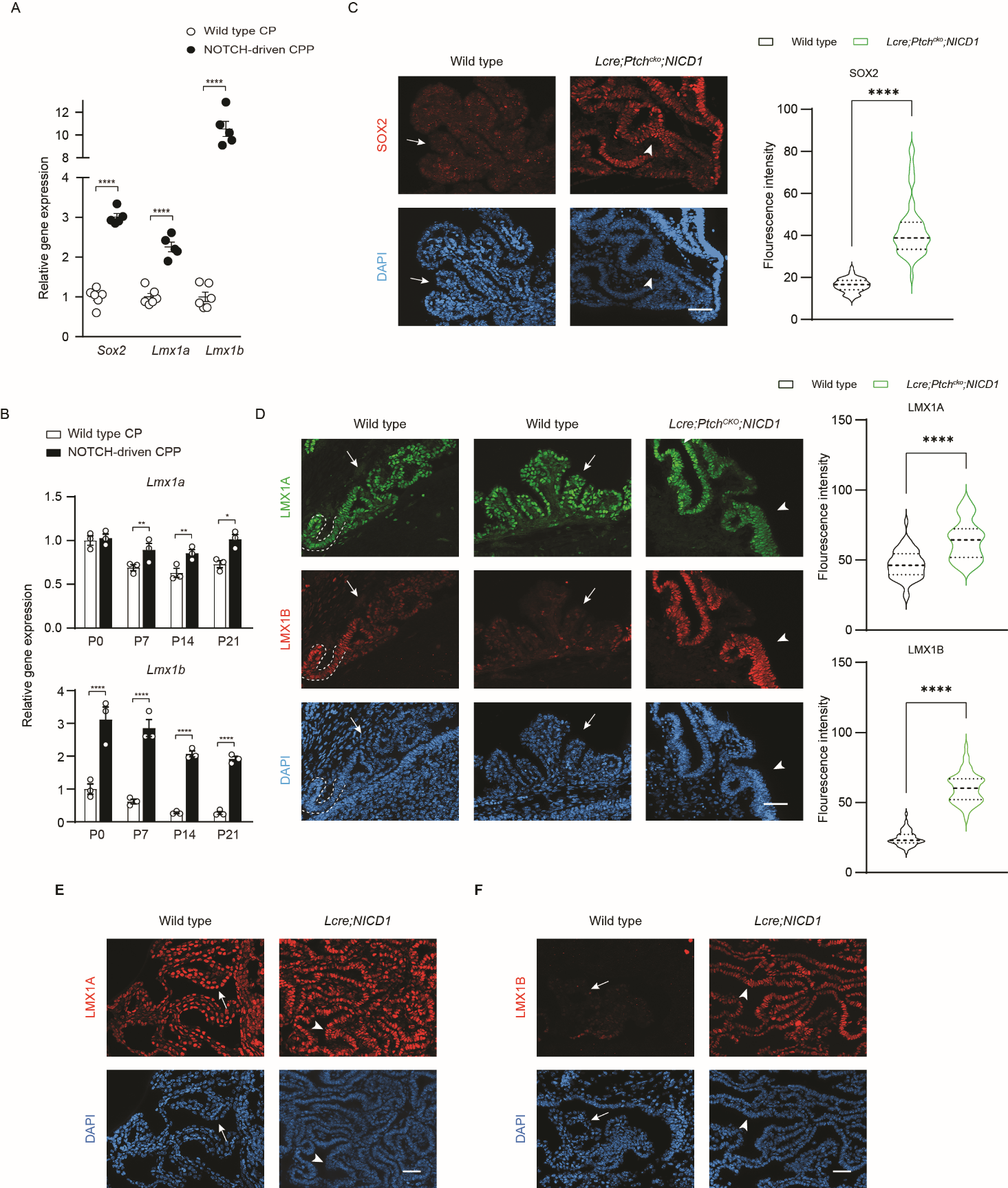
**

**Figure S5. Increased expression of LMX1A and LMX1B in NOTCH-driven CPP and CPC, related to Figure 5.** (A, B) RT-qPCR analysis of the expression of *Sox2, Lmx1a*, *and Lmx1b* in CP in wild type mice, and NOTCH-driven CPP in *Lcre;NICD1* animals (*n* = 5 per genotype [A], *n* = 3 per time point per genotype [B], mean ± s.e.m., two-tailed unpaired *t*-test, * < 0.05; ***P* < 0.01; *****P* < 0.0001). Three independent experiments were conducted. (C, D) Immunofluorescence of SOX2 (C, red), LMX1A (D, green), and LMX1B (D, red) is shown in upper rhombic lip/roof plate (marked by dotted lines), and the CP (arrows) in wild type mice, and CPC (arrowheads) in *Lcre;Ptch^cko^;NICD1* animals at day E14.5. DAPI (blue) labels nuclei. Scale bar, 50µm. Fluorescence intensity of SOX2 (C), LMX1A/B (D) in upper rhombic lip/roof plate is quantified (*n* = 50, mean ± s.e.m., two-tailed unpaired *t*-test, *****P* < 0.0001). The experiments were repeated three times independently. (E, F) Immunofluorescence of LMX1A (E, red) and LMX1B (F, red) is shown in the hindbrain CP in wild type animals, and CPP in *Lcre;NICD1* animals. Arrows point to CP, while arrowheads point to CPP. DAPI (blue) labels nuclei. Scale bars, 50µm. Three independent experiments were conducted.

**
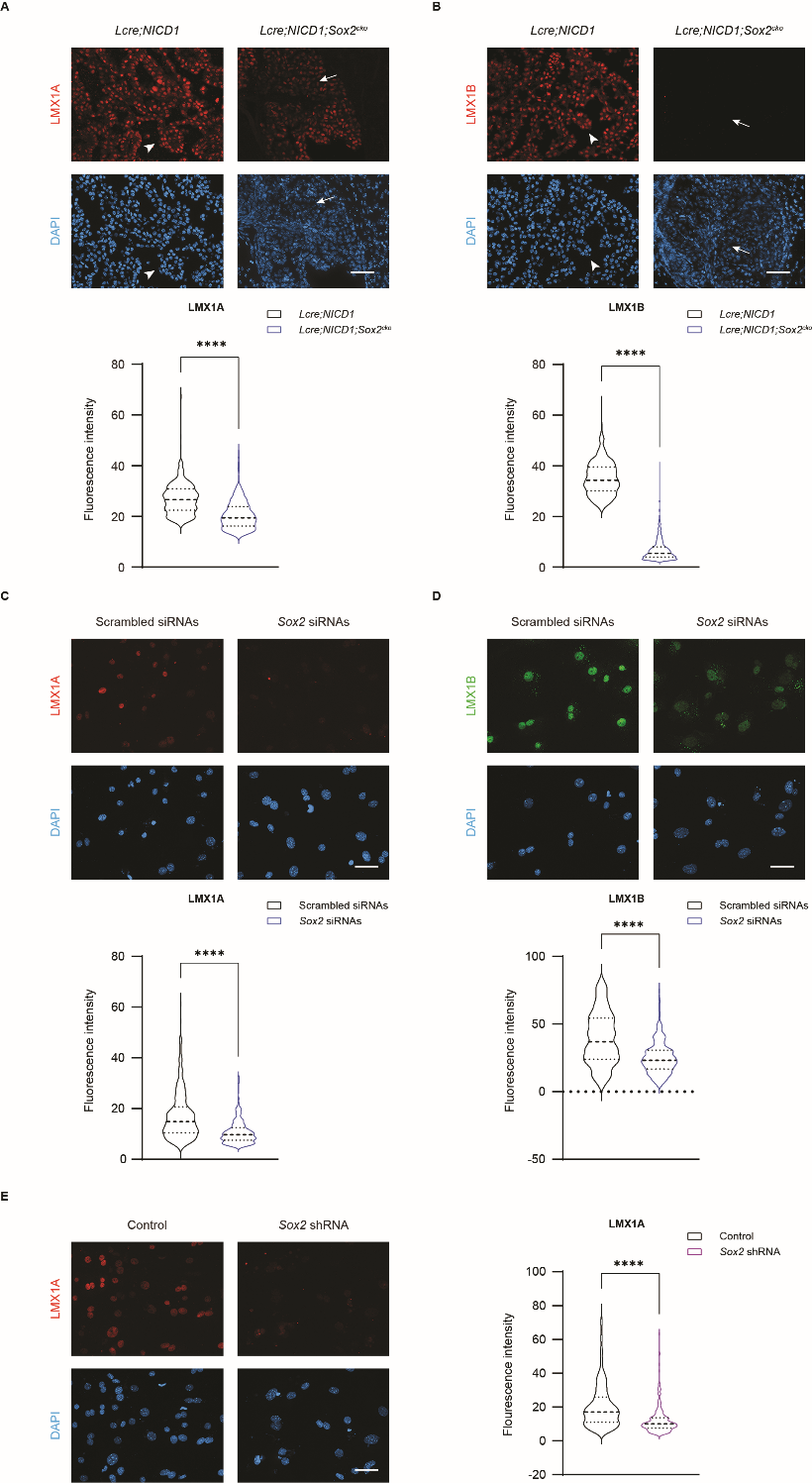
**

**Figure S6. *Sox2* regulates LMX1A and LMX1B transcription factors in NOTCH-driven CPP, related to Figure 5.** (A, B) Immunofluorescence of LMX1A (A, red) LMX1B (B, red) is shown in CPP in *Lcre;NICD1* and *Lcre;NICD1;Sox2^cko^* animals at day P18. Arrowheads point to tumor cells, arrows point to SOX2-deficient tumor cells. DAPI (blue) labels nuclei. Scale bars, 50µm. Fluorescence intensity of LMX1A and LMX1B is quantified (*n* = 370 [LMX1A], *n* = 747 [LMX1B] for CPP cells; *n* = 294 [LMX1A], *n* = 662 [LMX1B] for SOX2-deficient tumor cells; mean ± s.e.m., two-tailed unpaired *t*-test, *****P* < 0.0001). The experiments were repeated three times independently. (C, D) Immunofluorescence of LMX1A (C, red) or LMX1B (D, red) is shown in NOTCH-driven CPP cells treated with control scrambled siRNA or a pool of siRNAs against *Sox2*. DAPI (blue) labels nuclei. Scale bars, 50µm. Quantification of fluorescence intensity of LMX1A and LMX1B is shown (control siRNAs: *n* = 353 [LMX1A], *n* = 354 [LMX1B]; *Sox2* siRNAs: *n* = 372 [LMX1A], *n* = 366 [LMX1B]; mean ± s.e.m., one-way ANOVA, *****P* < 0.0001). Three independent experiments were conducted. (E) Immunofluorescence of LMX1A (red) is shown in NOTCH-driven CPP infected with control viruses or viruses expressing shRNAs against *Sox2*. DAPI (blue) labels nuclei. Scale bar, 50µm. Fluorescence intensity is quantified (control: *n* = 477 cells; *Sox2* shRNAs: *n* = 266; mean ± s.e.m., two-tailed unpaired *t*-test, *****P* < 0.0001). Three independent experiments were conducted.

**
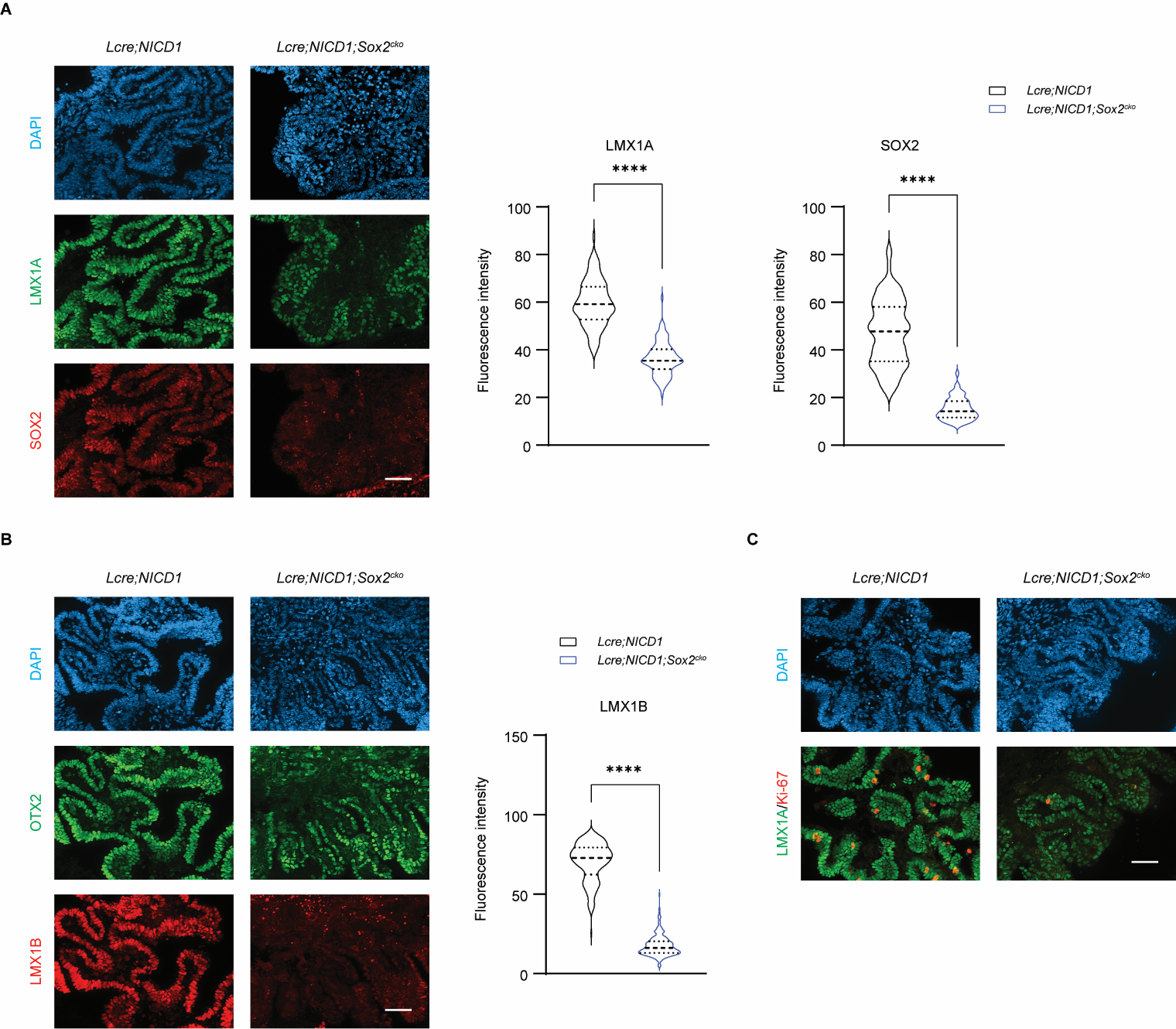
**

**Figure S7. SOX2 regulates LMX1A and LMX1B expression in NOTCH-driven CPP during development,** **related to Figure 5.** (A) Immunofluorescence of LMX1A (green) and SOX2 (red) is shown in CPP in *Lcre;NICD1* and *Lcre;NICD1;Sox2^cko^* animals at day E13.5. DAPI (blue) labels nuclei. Scale bar, 50µm. Quantification of fluorescence intensity of LMX1A and SOX2 is shown (*n* = 200 [SOX2], *n* = 100 [LMX1A] for CPP cells; *n* = 111 [SOX2], *n* = 100 [LMX1A] for SOX2-deficient tumor cells; mean ± s.e.m., two-tailed unpaired *t*-test, *****P* < 0.0001). Results were obtained from three independent experiments. (B) Immunofluorescence of LMX1B (red) and OTX2 (green) is shown in CPP in *Lcre;NICD1* and *Lcre;NICD1;Sox2^cko^* animals at day E13.5. DAPI (blue) labels nuclei. Scale bar, 50µm. LMX1B fluorescence intensity is quantified (*n* = 200 for CPP cells; *n* = 201 for SOX2-deficient tumor cells; mean ± s.e.m., two-tailed unpaired *t*-test, *****P* < 0.0001). Three independent experiments were conducted. (C) Immunofluorescence of LMX1A (green) and Ki-67 (red) is shown in CPP in *Lcre;NICD1* and *Lcre;NICD1;Sox2^cko^* animals at day E13.5. DAPI (blue) labels nuclei. Scale bar, 50µm. Results were obtained from three independent experiments.

**
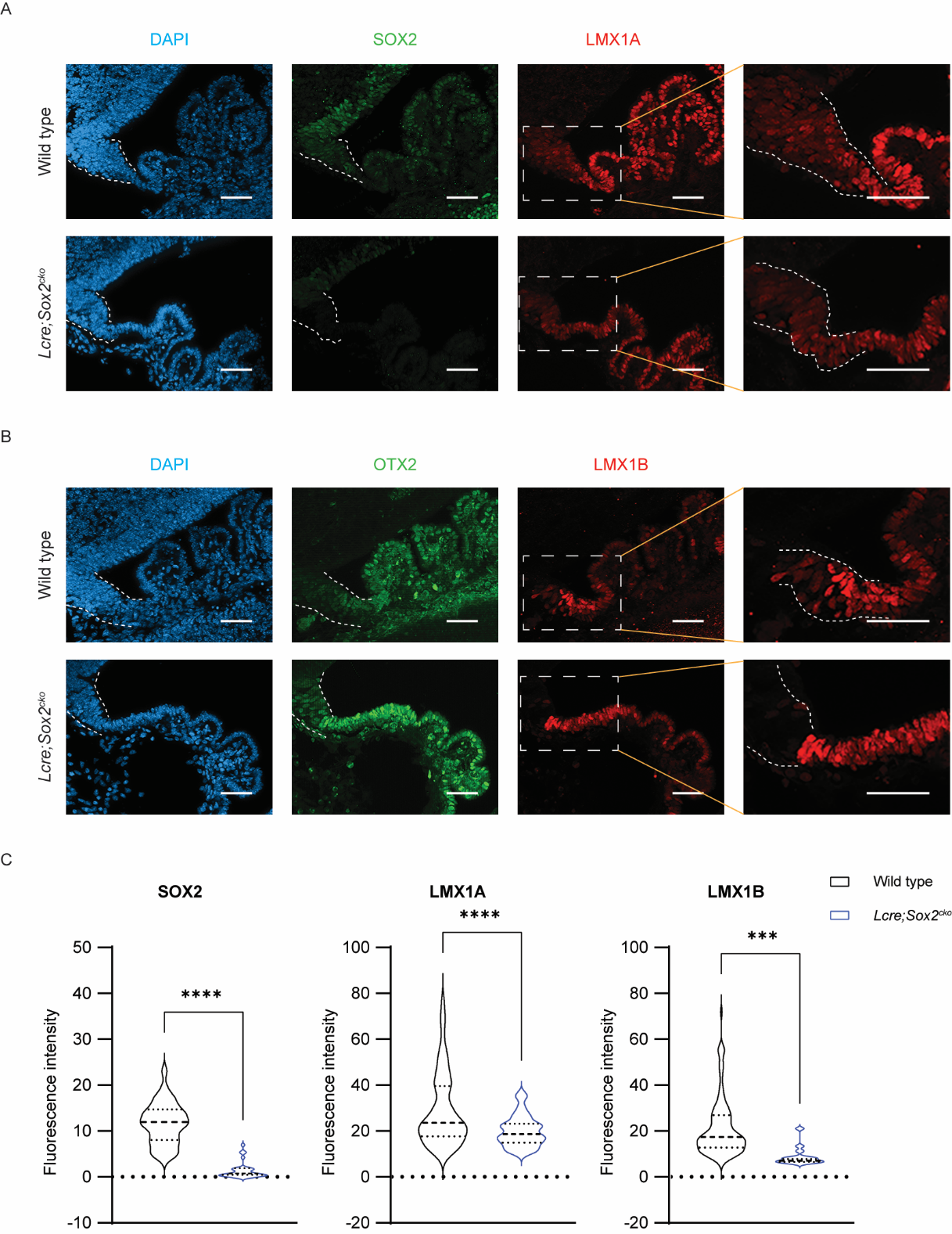
**

**Figure S8. SOX2 regulates LMX1A and LMX1B expression in the rhombic lip/CP during development, related to Figure 5.** (A) Immunofluorescence of SOX2 (green) and LMX1A (red) is shown in upper rhombic lip/roof plate (marked by dotted lines) in wild type and *Lcre;Sox2^cko^* animals at day E14.5. Region in bracket is shown in higher magnification on the right. DAPI (blue) labels nuclei. Scale bars, 50µm. Three independent experiments were conducted. (B) Immunofluorescence of OTX2 (green) and LMX1B (red) is shown in upper rhombic lip/roof plate (marked by dotted lines) in wild type and *Lcre;Sox2^cko^* animals at day E14.5. Region in bracket is shown in higher magnification on the right. DAPI (blue) labels nuclei. Scale bars, 50µm. Three independent experiments were conducted. (C) Fluorescence intensity of SOX2 and LMX1A in (A), and LMX1B in (B) in upper rhombic lip/roof plate is quantified (wild type cells: *n* = 116 [SOX2], *n* = 113 [LMX1A], and *n* = 100 [LMX1B]; cells from *Lcre;Sox2^cko^* animals: *n* = 63 [SOX2], *n* = 59 [LMX1A], and *n* = 50 [LMX1B]; mean ± s.e.m., two-tailed unpaired *t*-test, *****P* < 0.0001). The experiments were repeated three times independently.


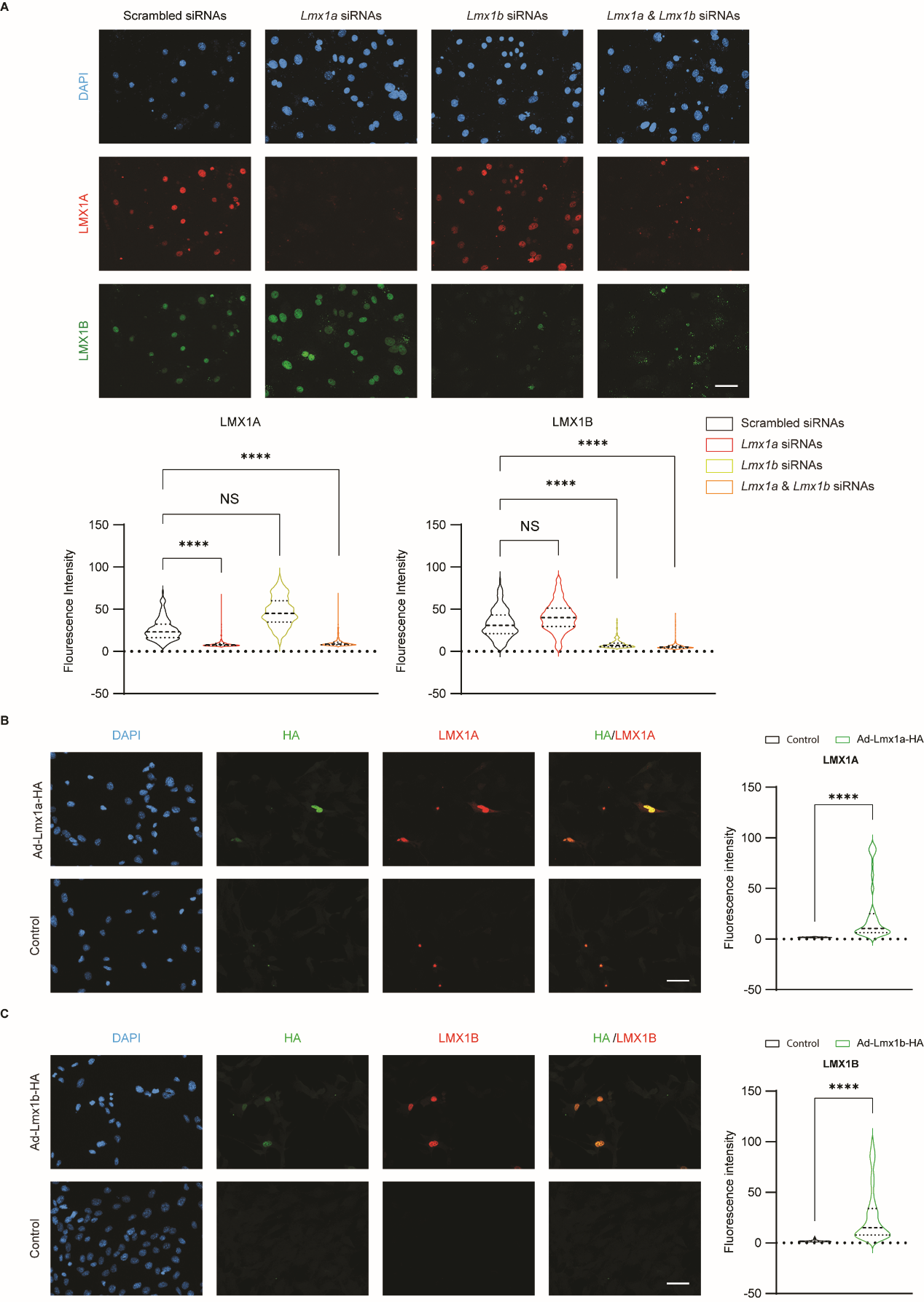


**Figure S9. Knockdown and overexpression of LMX1A and LMX1B transcription factors in CP tumor cells, related to Figure 6.** (A) Immunofluorescence of LMX1A (red) and LMX1B (green) is shown in NOTCH-driven CPP treated with control scrambled siRNAs, or pools of siRNAs against *Lmx1a* and/or *Lmx1b*. DAPI (blue) labels nuclei. Scale bar, 50µm. Fluorescence intensity of LMX1A and LMX1B is quantified (*n* = 336 cells per group [Control siRNAs]; *n =* 610 cells [*Lmx1a* siRNAs], *n =* 283 cells [*Lmx1b* siRNAs], *n =* 886 cells [*Lmx1a* & *Lmx1b* siRNAs]; mean ± s.e.m., one-way ANOVA, *****P* < 0.0001; NS, non-significant). Three independent experiments were conducted. (B, C) Immunofluorescence of LMX1A (B, red), LMX1B (C, red), and hemagglutinin (HA, green) is shown in CPC cells infected with control viruses, or viruses expressing HA-tagged LMX1A or LMX1B in *Rb1/Trp53*-deficient CPC cells. DAPI (blue) labels nuclei. Scale bars, 50µm. Fluorescence intensity is quantified (cells infected with viruses: *n* = 369 [LMX1A], *n* = 206 [LMX1B]; control cells: *n* = 159; mean ± s.e.m., two-tailed unpaired *t*-test, *****P* < 0.0001). Results were obtained from three independent experiments.
